# Re-thinking the Migration of Cariban-Speakers from the Middle Orinoco River to North-Central Venezuela (AD 800)

**DOI:** 10.1007/s10963-017-9102-y

**Published:** 2017-05-05

**Authors:** Andrzej Antczak, Bernardo Urbani, Maria Magdalena Antczak

**Affiliations:** 1 Leiden University, Leiden, The Netherlands; 2 Universidad Simón Bolívar, Caracas, Venezuela; 3 Instituto Venezolano de Investigaciones Científicas, Caracas, Venezuela

**Keywords:** Cariban-speakers, Arawakan-speakers, Archaeo-linguistics, Arauquinoid culture, Valencioid culture, Venezuelan archaeology, Lowland South American archaeology

## Abstract

Moving back in time from the early colonial to the late pre-colonial period we evaluate the hypothesis asserting the migratory movement of Cariban-speaking groups from the Middle Orinoco River area towards north-central Venezuela. The explanation in vogue maintains that the migration followed fluvial routes and occurred between 1350 and 1150 BP (AD 600–800). We examine archaeological, linguistic, ethnohistorical, genetic, and ecological data seeking similarities between the Orinoco emigrants and their north-central Venezuelan descendants. As a result, we propose an alternative terrestrial/fluvial route and suggest these events occurred between 1150 and 1050 BP (AD 800–900). The route first proceeded upstream along rivers of the central *llanos* and later followed a natural terrestrial geomorphological corridor into the Lake Valencia Basin. We argue that, while future interdisciplinary (especially archaeo-linguistic and bioarchaeological) research is needed to further assess the results of these analyses, the Orinocan descendants in north-central Venezuela emerge as one of the most dynamic sociopolitical Cariban-speaking entities in all northeastern South America and the insular Caribbean on the eve of the European Conquest.

## Introductory Remarks

One of the fundamental problems confronted by archaeology has been whether the similarities observed in material culture found in two different geographical regions are the result of shared ancestry, demic or cultural diffusion, or convergent adaptation to similar selective pressures (Fort et al. 2015). Historical linguists and archaeolinguists face a further challenge when trying to synchronize the archaeological evidence and linguistic features of two geographic regions in a historical perspective. Both problems are best approached through interdisciplinary cooperation among the fields of computer science, bioinformatics, anthropology, archaeology, history, genetics, and the life sciences (McConvell 2010; Koch et al. 2014; Heggarty 2015). In this paper, we bring together sources of data from diverse disciplines in order to reconstruct the past sociocultural landscapes in which the similarities observed in the archaeological record between the Middle Orinoco and north-central Venezuela have traditionally been attributed to demic diffusion of indigenous peoples (Kidder 1944; Cruxent and Rouse^1958^; Rouse and Cruxent 1963). This migration from the Middle Orinoco to the north has been conventionally associated with the concomitant spread of Cariban languages (Zucchi 1978; Zucchi and Denevan 1979; Tarble and Zucchi 1984; Tarble 1985). Although some of the causes, timing and direction of this migration have been identified in the mid-twentieth century, this occurred using an outdated monolithic conception of archaeological cultures that largely conflated biological signatures of human populations with their cultural identities and languages, privileging long-range migrations of indigenous peoples over local developments (Osgood and Howard 1943; Rouse 1951; Wagner 1978). Our discussion of the data drawn from diverse sources focuses on the interrelated processes of culture and language change that might have occurred in concrete sociocultural landscapes of the pre-colonial past. By exploring and hypothetically interconnecting novel interdisciplinary scenarios, we aim to challenge in-vogue ‘truths’ and reinvigorate this old debate which remains of great relevance to the archaeology of northeastern South America.

## Early Conceptualizations of Venezuelan Archaeology

Since the early twentieth century, Venezuelan archaeology has been considered pivotal in understanding the movements of indigenous peoples, goods and ideas between the Andean west, the Caribbean north, and the Tropical Lowland south. In 1916, Herbert Spinden posited the ‘intermediate role’ of Venezuela in the archaeology of northern South America (Spinden 1916). In order to support this hypothesis, he not only carried out the pioneering reconnaissance of northern and central Venezuela but also examined private collections and even analyzed petroglyphs and pictographs. Spinden argued that connections between the archaeology of Venezuela and the neighboring regions attributed to ‘cultural dissemination’ could be observed in the morphology and decorations of ceramic wares and figurines, as well as in the tradition of urn burial that extended beyond Venezuela to Brazilian Guiana, the Island of Marajó in the mouth of the Amazon, and to Nicaragua to the northwest (Spinden 1916, p. 327).

Osgood and Howard (1943), the authors of a preliminary—but the first nationwide—archaeological survey, indicated Venezuela as a region of great archaeological importance in interlacing diverse macroregional cultural influences. They compared its position to the horizontal bar of a letter H that connects the right vertical bar, representing indigenous migration routes spreading along the eastern part of South America and out through the insular Caribbean, to the left vertical bar representing migrations between Mesoamerica and the Andes via Central America. Julian Steward (1948), for his part, perceived parallels in sociopolitical, material and religious developments in the ‘circum-Caribbean culture area’ in which he included Central America, Colombia, Venezuela, and the Caribbean (see Willey 1960). The archaeological cultures from the Lake Valencia Basin and the Middle Orinoco that are the focus of this paper have been considered the best examples of influences coming from both the highland and lowland regions (Navarette 2008, p. 429; see also Gassón and Wagner 1994, 1998).

In their seminal chronological outline of the archaeological cultures of Venezuela, Cruxent and Rouse (1958, vol. 1, p. 21) went further than their predecessors by indicating that the geographical position of Venezuela ‘offered optimal conditions for migratory movements’ not only for humans but for plants and animals. Moreover, they rejected the portrayal of Venezuela as an inert backdrop against which pre-Columbian peoples performed migratory movements following unilinear flows. Instead, they envisioned multidirectional and multiple lines of migration and diffusion (Cruxent and Rouse 1958, p. 22). They also pointed to the active contribution by Venezuelan cultures to the origin and development of the above-mentioned west–east dichotomies (Rouse and Cruxent 1963, p. 6). However, despite such outstanding insights, practitioners of the culture-historical approach, including Cruxent and Rouse, remained convinced that similar ceramic attributes directly reflect shared cultural norms. While operationalizing this paradigm, little attention was paid to the question of how the fabrication and use of pottery both reflected and formed their makers’ notions of sameness and affinity (Sørensen-Stig 2014, p. 256). Meanwhile, non-ceramic remains and features largely lie outside the area of immediate interest (Cruxent and Rouse 1958, vol. 1, pp. 23–24), further limiting access to the socio-material and ideological aspects of the Amerindian past (Antczak and Antczak 2016). The normative approach to culture imposed a straitjacket hindering novel conceptualizations of intersocietal interactions as well as interrelations between archaeological remains, Amerindian mobility, and historical-comparative linguistics (Hofman and Carlin 2008).

In the late 1960s and early 1970s the onset of Venezuelan social archaeology, based on the principles of historical materialism (e.g. Sanoja and Vargas-Arenas 1974; Vargas-Arenas 1998), created a singular blend of general Marxist principles with elements of environmental materialism (Navarette 2004, p. 232). Even if this approach aimed at linking the indigenous past with the present and invoked internal political and socio-economic developments rather than demic or cultural diffusion as the causes of the archaeologically observed shifts, its early constructs still heavily depended on archaeological evidence compiled by Cruxent and Rouse (1958; Rouse and Cruxent 1963). It is in the above-outlined context of theory and practice in Venezuelan archaeology that the central concern of this paper began to take shape.

## Demic Diffusion and Language ‘Spread’

The search for correlations between archaeological evidence (still mainly ceramics) and linguistic phenomena in the study region began in the wake of Donald Lathrap’s seminal work (1970). His focus was on the macroregional scale, encompassing the Andes as well as the Amazonian and Orinoquian lowlands, in addition to the insular Caribbean (Zucchi 1984; Arvelo and Wagner 1984; Rouse 1985; Molina 1985; Meggers 1987; Oliver 1989). In Venezuela, retaining the main culture-chronological units conceptualized by Cruxent and Rouse (1958), the aim was to understand by means of migration studies the pre-Hispanic cultural interrelations among increasingly smaller (archaeologically speaking) regional units. This trend marked the onset of attention to the archaeo-linguistics of migration in the study area. In two of these studies, Zucchi (1978, pp. 356–357) and Tarble (1985), proposed that the expansion of presumably Cariban-speakers from the Middle Orinoco River area to north-central Venezuela between 1350 and 1150 BP (AD 600–800) was linked to population growth in their ancestral settlements along the banks of this major waterway. Lathrap (1970) also suggested that the best areas for human occupation in the lowlands were the seasonally flooded plains along the Amazon and Orinoco Rivers (Denevan 1996). Therefore, it has been further argued that because these areas were limited, population pressure forced residents to migrate towards other environments, such as seasonally flooded savannas (Gassón 2002, p. 294). In the Middle Orinoco region, the socio-cultural growth on riverbanks furthered: (1) demographic saturation; (2) the introduction of new agricultural techniques; (3) expansion towards adjacent areas; and (4) further migration to areas distant from the Orinoco (Tarble 1985). These factors—coupled with a probable increase in the frequency and intensity of interactions related to exchange as well as in violent confrontations with competing groups, mainly Arawakan language speakers—could have driven Cariban-speakers from the Middle Orinoco west, east and north (Zucchi and Denevan 1979; Roosevelt 1980; Zucchi 1985, 2002).

The expansion of the Cariban-speakers identified with Arauquinoid ceramic complexes towards the northeast reached as far as Trinidad (Boomert 2000), the Guianas, and Surinam (Boomert 1980; Rostain and Versteeg 2004; Rostain 2008, 2012a; Versteeg 2008). These migrants were related to the makers of Valloid pottery from the Middle Orinoco region dated to between 1455 ± 140 (AD 495; GX-8981) and 300 ± 140 BP (AD 1650; GX-8985) and associated with the Western Guiana Cariban linguistic subgroup (Tarble and Zucchi 1984, p. 442, Table 2). The Arauquinoid from the Orinoco and the Guianas expanded south as well into the Middle–Lower Amazon between 1450 and 950 BP (AD 500–1000), as inferred from the presence of ‘incised-punctate’ pottery associated with the Santarém or Tapajônica archaeological culture (Heckenberger and Góes Neves 2009, p. 256; Cavalcante-Gomes 2002). Zucchi (1990) also proposed that multiethnic groups composed of Arawakan-speaking Cedeñoid and Cariban-speaking Arauquinoid-Valloid pottery makers migrated first to the northern Caribbean coast of Venezuela and from there to Santo Domingo between 1250 and 1150 BP (AD 700–800). Recently van den Bel (2015, p. 596) questioned the migration of Arauquinoid peoples to the coastal Guianas, suggesting that contact and exchange may well account for the archaeological evidence.

Critics of the population pressure model have demonstrated that it is not supported by archaeology, ethnohistory, or ethnographic analogy (Denevan 2001; Gassón 2002) and that such pressure is not needed to produce inter- and intra-ethnic stratification (Heinen and García-Castro 2000). However, all these theoretical considerations left intact the archaeological evidence that has traditionally been considered proof of Arauquinoid spatial expansion from the Middle Orinoco to north-central Venezuela.

## The Archaeological Regions Under Study

The archaeological regions of the Middle Orinoco and north-central Venezuela are separated by a straight line extending some 300 km. The former region has been situated by most Orinoquian archaeologists between the modern cities of Puerto Ayacucho (Amazonas State) and Ciudad Bolívar (Bolívar State). This is one of the main regions of the Orinoco River Basin, the watershed of the 2200 km waterway (Gassón 2002, p. 239). Vast areas adjacent to the banks of the river there are flooded annually during the rainy season. Although these floods make the area hazardous for settlers, they also create environs relatively rich in biomass and resources, especially in the middle section of the river.

The region of north-central Venezuela, located in northeastern South America, encompasses the modern cities of Valencia, Maracay, and Caracas. It is comprised, north to south, of (1) offshore islands and archipelagos; (2) the Caribbean coast and high-elevation tropical cloud forest in the Cordillera de la Costa (the highest peak reaches 2765 masl); and (3) the Lake Valencia Basin. South of the lake rises the Cordillera del Interior mountain range which, at an average altitude of 800 masl, forms the northern boundary of an area containing extensive *llanos* (savanna plains and seasonal wetlands) and the Orinoco River further south (Antczak and Antczak 2006).

## Methods and Sources of Data

This paper utilizes previously published data while also mobilising new evidence drawn from archaeology, ethnohistory, linguistics, genetics, and ecology. We begin with the early sixteenth century record of the historically known Cariban-speaking †*Caraca* Indians from north-central Venezuela and their western neighbours, the Arawakan-speaking †*Caquetío*. To go back beyond the year 1498 (the year Columbus voyaged to northeastern Venezuela), we here employ the concept of direct historical approach in its broad meaning, which oriented the scholarly search for direct historical connection between ethnographically documented and archaeological cultures (see Lyman and O’Brien 2001). Operationalizing this concept, we explore the cultural continuity between the above-mentioned historically known groups and their immediate predecessors, that is, the bearers of the Valencioid and Dabajuroid archaeological cultures, respectively. Going further, we review the available data provided by archaeology, landscape engineering, genetics, and chronology to test the claim for close relations between the Valencioid culture of north-central Venezuela and the Arauquinoid culture of the Middle Orinoco region. We propose an alternative route and timing of the northward migration to those that are currently in vogue. Continuing with the direct historical approach, we aver that the compiled data seems to support the previous claim about linguistic continuity among the Arauquinoid migrants, their Valencioid successors, and the linguistic affiliation of Amerindian groups documented in north-central Venezuela in the early sixteenth century. Finally, we present our conclusions and indicate avenues for future research.

### Cariban-Speakers in North-Central Venezuela

By the mid sixteenth century, Amerindian populations spoke Cariban languages along Venezuela’s Caribbean coast from the Paria Peninsula in the east to what is presently known as the Central Coast in the west (Loukotka 1968; de Civrieux 1980; Durbin 1985; Migliazza 1985; Villalón 1987; Antczak 1999; Biord 2001; Gildea 2003). These languages were recently grouped into the Venezuelan Branch of Carib languages (Gildea 2012). Most probably, the Cariban-speaking societies participated in a larger coastal interethnic system of interdependence which might have been linked to a similar system in the Middle and Lower Orinoco region (Biord 1985, 2005; Arvelo-Jiménez et al. 1989; Arvelo-Jiménez and Biord 1994; Tiapa 2007; Morales Méndez 2007). Durbin has suggested that proto-Cariban speakers dispersed from an ancestral homeland in the Guianas, where the highest concentration of Cariban-speaking groups was found. According to lexical-statistical data, proto-Cariban began to diverge from the common trunk some 4400 years ago (Layrisse and Wilbert 1966). Oliver (1989, p. 225), based on linguistic and archaeological evidence, suggested that the Coastal Caribs diverged from the branch of the Northern Caribs about 2300 years ago. The only Cariban language that was grouped as Coastal but was also spoken outside the Caribbean coastal area was †*Tamanaco*, reported in the sixteenth–eighteenth centuries in the area of the Middle Orinoco (Matteí-Müller and Henley 1990, p. 34).

It has been suggested that the Cariban-speaking makers of the Arauquinoid/Valloid pottery from the Middle Orinoco region migrated north and settled in the Lake Valencia Basin, giving birth to the Valencioid culture (Cruxent and Rouse 1958; Sanoja and Vargas-Arenas 1974, 1999; Tarble 1985; Zucchi 1985; Tarble and Zucchi 1984; Vargas-Arenas 1990). Upon their arrival, the immigrants entered into interaction with the locally established populations, the Barrancoid/Saladoid pottery makers in the Lake Valencia Basin and the Ocumaroid along the coast. The Ocumaroid were purportedly Arawakan-speakers and their pottery emerged through a process of sociocultural interactions that blended tangible and intangible traits of Archaic Age, Tocuyanoid, Saladoid, and Barrancoid traditions, somewhere after 1750–1650 BP (AD 200–300) (Sýkora 2006; Rivas 2001; Antczak and Antczak 2006). It is noteworthy that, contrary to scholars who supported the hypothesis of the Cariban-speaking origin of the Valencioid culture bearers, Alfredo Jahn (1932, pp. 4–7) suggested that Arawakan-speaking Valencioids were overwhelmed by Cariban-speakers only shortly before the European Conquest. Strong but unspecified external cultural influence reaching the Lake Valencia Basin just before the European Conquest was a motif that reappeared in several later writings on regional archaeology (e.g. Bennett 1937, p. 89; Peñalver 1976). These hypotheses, largely based on intuition, require close attention in future research.

From an archaeological perspective, the late pre-Hispanic inhabitants of north-central Venezuela were integrated within the Valencioid Sphere of Interaction as defined by Antczak and Antczak (1999; see also Guzzo Falci et al. 2017; Laffoon et al. 2016). They were associated with the western segment of northern Cariban-speakers, and linked to the protohistoric †*Caraca* Amerindians (Antczak 1999; Biord 2001). The Valencioid Sphere of Interaction was initially defined on the basis of the spatial dispersion of pottery which presents the stylistic features of the Valencioid series (Cruxent and Rouse 1958). In chronological terms, the sphere was purportedly operational between 750 BP (AD 1200) and the advent of the European conquest. At this time, many cultural changes were occurring throughout the South American Lowlands. In the Guianas, for instance, there was displacement of Koriabo groups and, at the same time, the demise of Arauquinoid raised-field agriculture began. These phenomena might have been related to some little-known macroregional climatic changes (Rostain 2008, 2012b).

**Fig. 1:**
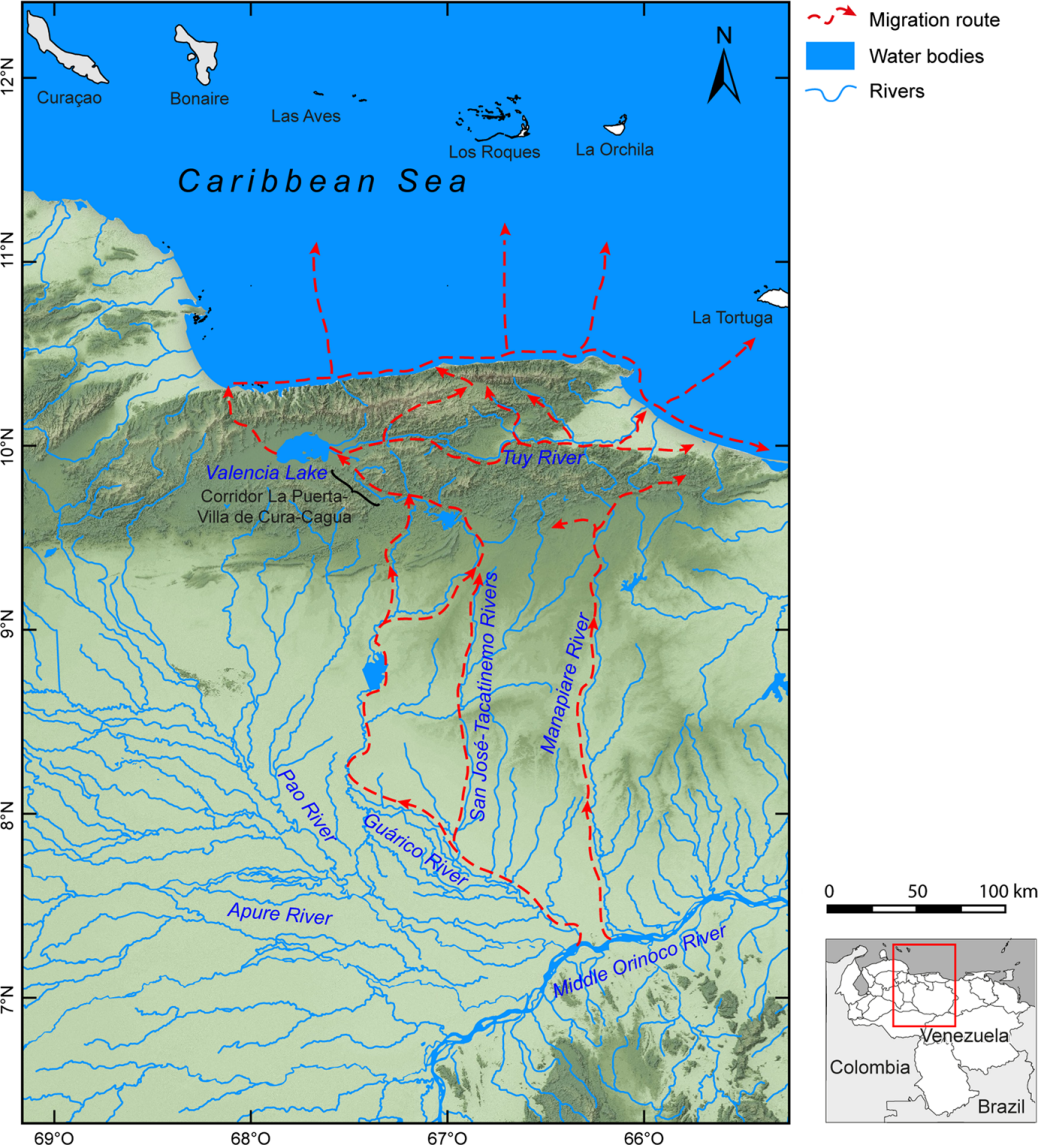
Suggested migratory routes of the Carib-speakers with respect to the geomorphology and the topography of the macro-region

Geographically, the sphere extended across a wide area of outstanding geomorphologic, topographic and bio-ecologic diversity, including, from north to south, the oceanic islands of Los Roques and La Orchila, the mainland coast between Tucacas and Cape Codera, the Cordillera de la Costa mountain range with its cloud forests, the Lake Valencia Basin, the valleys of the Aragua, Caracas, and Tuy Rivers, and the surrounding mountainous areas (Fig. 1). The centre of the sphere, from which the Valencioid culture began to radiate increasingly after 1150–1050 BP (AD 800–900) through mobility and exchange, was situated in the Lake Valencia Basin (Antczak and Antczak 1999). The main Valencioid settlements were concentrated on the fertile floodplains abutting the eastern and western shores of Lake Valencia. There, several complexes of earthen mounds were constructed on top of which the Valencioids built houses, planted, and buried their dead (Osgood 1943; Bennett 1937; del Valle and Salazar 2009; Tarble 1986). The economy was based on hunting, gathering, lacustrine fishing, and horticulture. Village stability and population growth were purportedly direct consequences of the horticultural efficiency achieved on the seasonally flooded shores of Lake Valencia. Seeking to complement the locally available resources with those of different ecological zones, the purported Valencioid chiefdom expanded both directly (through colonization of new lands) and indirectly (through subjugation of the egalitarian peripheral societies) (Sanoja and Vargas-Arenas 1974; Vargas-Arenas 1985; Vargas-Arenas 1990). The hypotheses above remain in need of further testing (Antczak and Antczak 2006). Archaeological evidence for intense horticulture is null in the otherwise haphazard environment on the shores of Lake Valencia (see Jahn 1940). It is equally absent for the purported violent subjugation of neighbouring societies to the north. Evidence of burnt villages, violent skeletal trauma and population displacement is lacking, while control of regional chronology is extremely weak (Antczak and Antczak 1999).

Antczak and Antczak (2006) presented an alternative perspective on the Valencioid trajectory, arguing that after a few centuries of development, largely within the confines of the Lake Valencia Basin (1150–850/750 BP [AD 800–1100/1200]), factions of Valencioid society migrated north to the coast and entered into direct inter-lingual interaction (intermarriage, cohabitation, joint fishing expeditions, ritual assistance) with the purportedly Arawakan-speaking Ocumaroid. The linguistic correlate to the archaeologically-defined Ocumaroid culture is hypothetically based on an assumption that the Ocumaroid ceramic series (as defined by Rouse and Cruxent 1963) might have been the ‘result’ of incursions of Arawakan-speaking Saladoid from eastern Venezuela onto the Caribbean coast, and their subsequent interactions with the local Archaic Age populations and Tocuyanoid culture-bearers of the north-central coast, during the first centuries AD (see Sýkora 2006). The migration of the factions of the inland Valencioid, the traditional makers and users of an unpainted but richly modelled pottery, and their cohabitation on the coast with the Ocumaroid producers of painted wares, gave birth to the transcultural ceramic assemblage that combined both plastic and painted decorations. Resulting hybrid wares may be seen as both the material embodiment and, concomitantly, the means of furthering new identities, ideologies, and practices that might have been evolving in these intersocietal, intercultural, and interlingual engagements (see DiPaolo Loren 2015, p. 6). Herrera-Malatesta (2011) proposed the inclusion of late coastal ceramics in the Valencioid plastic/painted subseries, ascribing to it a temporal range of between 850 and 350 BP (AD 1100–1600).

During the last three centuries before the European Conquest, both the Valencioid and Ocumaroid commenced large-scale exploitation of marine resources on the offshore oceanic islands of the Los Roques Archipelago and brought to the mainland between three and five tons of queen conch meat annually (Antczak and Antczak 2006; Schapira et al. 2009). During this period the Valencioid Sphere of Interaction developed in north-central Venezuela. It was defined by a network of interactions that, operating through mobility and exchange mechanisms, fostered the social, economic, and ideological relations capable of supporting the Los Roques enterprise. But the fate of the Valencioid moundbuilders in the Lake Valencia Basin at the time of the European Conquest remains uncertain.

### Arawakan-Speakers in North-Central Venezuela

To the north of the Orinoco, early colonial sources documented the presence of Arawakan-speakers in northwestern and central Venezuela, along the maritime coast of the northeastern rim of the South American continent (Suriname, French Guiana, Guyana, and northeastern Venezuela), and in the Greater Antilles (Aikhenwald 1999, Table 3; Heckenberger 2002, map 4.1). Among the ethnohistorically better-known Arawakan speakers should be mentioned the †*Caquetío* people from the current state of Falcón lying on the west-central coast of Venezuela (Oliver 1989).

Moving back to pre-colonial times, the archaeo-linguistic hypotheses trace the origin of ethnohistorically known Arawakan-speaking peoples to the pottery-making horticulturalist groups from the Middle Orinoco area. From this perspective, the Middle Orinoco may be considered the point of departure for the dispersion of both Arawakan- and, later, Cariban-speaking peoples. The initial centre of dispersion of the Arawakan-speakers was, plausibly, located between the areas of the Upper Amazon River in Brazil and the Middle Orinoco River in Venezuela (Lathrap 1970; Oliver 1989; Rouse 1993; Zucchi 1991; Zucchi 2002; for the symbolic construction of indigenous regional landscapes see Ruette Orihuela 1998; Vall de la Ville 1998; Zucchi et al. 2001). The height of their territorial expansion was probably between 2450 and 1450 BP (500 BC–AD 500) (Heckenberger 2002, p. 99). By that time, the Middle Orinoco had become a homeland of archaeologically defined horticulturalists and makers of Saladoid and (purportedly) Barrancoid pottery, who were considered Arawakan-speakers (Gassón 2002). Currently, two main competing chronologies of Saladoid origin are in vogue (Gassón 2002, pp. 276–284): (1) a ‘long chronology’ that puts the date of the appearance of Saladoid white-on-red painted pottery at between 4450 and 2950 BP (2500–1000 BC) (Rouse 1978; Roosevelt 1987, 1997); and (2) a ‘short chronology’ that argues for Saladoid origins at the later date of 2600 BP (650 BC) and considers the painted pottery an intrusion (Sanoja and Vargas-Arenas 1974; Vargas-Arenas 1981, 1990).

From the Middle Orinoco area, the makers of Saladoid pottery moved down the Orinoco River, established permanent villages on the eastern coast of Venezuela, and from there moved along the coast and farther north toward the Antillean arc. The Barrancoid pottery makers supposedly followed their route. The archaeological signatures of their presence end on Trinidad, but the stylistic traits of Barrancoid pottery inspired the indigenous potters of the Lesser Antilles farther to the north (Boomert 2000). However, not all researchers agree that the Barrancoid culture originated in the Middle Orinoco region and that its bearers migrated downriver (see Boomert 2000 for summary of additional discrepancies with regards to the origin and spatial dispersion of Barrancoid pottery). According to Kidder (1944) and Rouse and Cruxent (1963), some of these groups migrated from the Middle or Lower Orinoco region towards the northwest to settle in the Lake Valencia Basin (at the Los Tamarindos site) and on the adjacent Caribbean coast at the El Palito site, between 1640 ± 120 BP (Y-579; AD 310) and 1615 ± 120 BP (Y-580; AD 335) (Rouse and Cruxent 1963, p. 155). Their materials were also recovered by Henriqueta Peñalver (del Valle and Salazar 2009) in the deeper (pre-Valencioid) layers of sites excavated around the Lake Valencia. Barrancoid pottery has also been reported from the mountains north of Lake Valencia (Antczak and Antczak 2007), further north on the Caribbean coast (Velázquez-Romero 2014), Valles del Tuy (Anonymous 1954), and in Caracas (Jam 1958; La Salle 2005) (see Table 2). However, as per the current research of José Oliver (2016, pers. comm.), Barrancoid complexes, strictly speaking, are not present in the Middle Orinoco region. The so-called Ronquín Sombra phase is, in essence, in the Saladoid series/tradition. Anna Roosevelt (1980), accommodating the archaeological evidence to Rouse and Cruxent’s theory of migration, suggested that the Ronquín Sombra phase signals a shift toward a more Barrancoid style. Thus far, however, there is no evidence in the Middle Orinoco of the emergence of a style/complex from the Ronquín Sombra phase that could correlate formally with the Barrancas (or pre-Classic Barrancas) that appear in the Lower Orinoco (J. Oliver 2016, pers. comm.). Yet, the ‘Barrancoid-like’ features (essentially modeled-incised decoration) could have resulted from various forms of interaction. Therefore neither hypothesis can be refuted, since the real conundrum is a still-unsolved problem of radiocarbon dates.

At the time of the Barrancoid arrival in north-central Venezuela the area was already occupied by a mosaic of human groups of diverse cultural origin and traditions, including most probably the remnants of Archaic Age populations (Antczak et al. 2017b). The presence of Saladoid pottery in north-central Venezuela and in the Lake Valencia Basin has also been pointed out (Cruxent and Rouse 1958, vol. 1, 161, 178; Arroyo 1999; Antczak 1999; Boomert 2000). However, no systematic study of the nature and dynamics of interaction between the Barrancoid and Saladoid groups, and other local residents has been undertaken (but see Sýkora 2006; Antczak and Antczak 2006). North-central Barrancoid pottery is morphologically akin to its Lower Orinoco counterpart, but at the same time shows elements of local continuity as well as simplification of forms and decoration. The presence of human figurines and ceramic pipes distinguishes it from the Orinoquian assemblages (Velázquez-Romero 2014). According to Rouse and Cruxent (1963, Fig. 17), the Barrancoid-style pottery makers could have survived on the Caribbean coast, in the coastal area of Taborda, until the time of the Spanish Conquest. Some cultural traits attributed to the Arawakan-speaking Saladoid-Barrancoid groups in the Middle and Lower Orinoco, the Lesser Antilles, and the central Amazon—traits such as the circular central plaza (Heckenberger 2002, p. 109) and characteristic village layouts (Siegel 1999)—remain undisclosed in the north-central Venezuelan region.

## Pondering Migrants and Immigrants

In the following sections, we discuss diverse categories of data that may be considered indicative of links between the bearers of Arauquinoid and Valencioid archaeological cultures.

### Mitochondrial DNA

Recently, Figuera-Pérez (2015) conducted research on Amerindian mitochondrial DNA persisting in current creole (*criollo*) populations of northern Venezuela. She found that mtDNA of Amerindian origin clearly differs between the northwestern population and the north-central/northeastern populations. Following Tarble and Zucchi (1984), Figuera-Pérez (2015, pp. 112–113) argued that such differences might be explained by cultural evidence found in the archaeological record. Although ethnicity cannot be determined using mtDNA, we would like to cautiously remark that the reported differences seem, geographically, to overlay the ethno-historical and linguistic distribution of Arawakan-speakers (†*Caquetío*) in northwestern Venezuela and Cariban-speakers (e.g. †*Caraca*, †*Teques*) in north-central and northeastern Venezuela as presented by Durbin (1977) (Fig. 4). In a similar vein, Castro de Guerra et al. (2009) discussed the implications of the study of Amerindian mtDNA in the current creole population of northwestern Venezuela for the understanding of pre-Hispanic migrations.

The increasing number of genetic studies in Venezuela, and particularly, the analyses utilizing next-generation sequencing (NGS) technologies, are welcome. They represent a novel and powerful tool for refining our understanding of whether specific archaeological traits spread across space due to demic or cultural diffusion. However, a sounder understanding of the diachronic configurations of the archaeological record across diverse sites and regions is necessary before genetic and cultural transmission can be temporally synchronized and meaningfully matched (Fort et al. 2015, p. 142). The archaeological data still hides a diversity of unspecified forms of interaction between local and non-local indigenous populations. Caution is also necessary when comparing Venezuelan data with genetic information coming from other parts of the Caribbean (Toro-Labrador et al. 2003; Carrero-González et al. 2010; Moreno-Estrada et al. 2013).

### Pottery

Alfred Kidder II (1944, p. 148) was the first to suggest a possible link between Valencioid (Valencia phase in Kidder 1948, pp. 420–424) and Arauquinoid pottery, indicating some stylistic features common to both assemblages. Rouse and Cruxent (1963) regarded the Valencioid series as a ‘degeneration’ of the Barrancoid series with the addition of stylistic Arauquinoid elements. Since then, isolated traits from the ceramic assemblages of Arauquinoid and Valencioid pottery have been compared to demonstrate a cultural relationship between these ceramic traditions. Below we discuss the form and decoration, temper, and the use of slip in both archaeological assemblages.

#### Plastic Decoration and Form

For Rouse and Cruxent (1963), decorative elements of the Arauquinoid and Valencioid assemblages have in common the coffee-bean eye, dotted eyebrows, and *adornos* with miniature features. Tarble and Zucchi (1984), in defining the Valloid series, also took note of these shared elements and added to the list dotted hubs (*mamelones*) and zoomorphic applications in the form of frogs (Arroyo 1999, p. 219). These elements have been reported across north-central Venezuela, including the archaeological sites in the Lake Valencia Basin (Marcano 1971a; von den Steinen 1904; Requena 1932; Bennett 1937; Osgood 1943; Kidder II 1944, Osgood and Howard 1943; Peñalver 1965, 1967, 1971); on the central-western coast (Morales 1984; Álvarez and Casella 1983; Martín 1995; Sýkora 2006; Herrera-Malatesta 2011); in the nearby Caracas Valley (Cruxent and Rouse 1958; Gómez-Aular 1995); and on the islands of the Los Roques Archipelago, as well as on La Orchila (Antczak and Antczak 1999, 2006). All of these sites fall within the Valencioid Sphere of Interaction defined by Antczak and Antczak (1999), and within the area of Cariban-speakers’ influence delimited by Durbin (1977).

Andrzej Antczak (1999) considered that comparative studies of Arauquinoid and Valencioid ceramics should also take into account the forms of vessels and the iconography used in their decoration. To demonstrate the potential of this proposition, Antczak (1993) analyzed the composition of a human face depicted on the neck of a ceramic jar dated to 580 ± 80 BP (AD 1370; I-16,323) and recovered in the Valencioid component at the Los Mangles site on La Orchila Island. The presentation of this face seems particularly similar to human faces applied to necked jars of composite silhouette excavated at the Corozal site in the Middle Orinoco area where these vessels had a long tradition (Camoruco Phases I–III, 1550–450 BP [AD 400–1500 based on radiocarbon date estimates and pottery seriation cited by Roosevelt 1980, Table 15, see also Figs. 69, 89]). The conclusion that the Los Mangles face is more closely related to Arauquinoid pottery (Cruxent and Rouse 1958, vol. 2, pl. 76, 1–4) than to depictions of the human face typical of the Valencia style gives rise to an interesting hypothesis. Antczak proposed that during late pre-colonial times, the Río Chico cultural area (on the continental coast south of La Orchila Island) might have been exposed to regular and direct Arauquinoid influences which were spreading directly from the Middle Orinoco through the eastern plains (see also Navarrete 2000; Rodríguez-Yilo 2001). The producers of Río Chico pottery would in that case have been the northern members of a wide interregional system linking the Middle Orinoco to the northeastern coast (Arvelo-Jiménez and Biord 1994). Simultaneously, they would have been a cog themselves in the northern coast sphere of interaction (Biord 2005). If so, then the pottery from the Los Mangles site might have been the result of persistent Middle Orinoco cultural influences on the Río Chico area. Antczak (1999) suggested that the decorated pottery associated with the date of 1820 ± 80 BP (AD 130; I-18,562) for the early strata of ceramic assemblage at the Los Cumaneces site on La Tortuga Island can be considered one of the earliest remains of Saladoid presence on the east-central Venezuelan coast. It would be even earlier than the El Mayal and Irapa Saladoid styles dated to 1795 ± 80 BP (AD 155; Y-297) and 1680 ± 85 BP (AD 270; Y-1113) (Rouse and Cruxent 1963, pp. 155–156), as well as El Cuartel pottery with minimum age of 1660 BP (uncertainty range not provided) (AD 290; IVIC-777) (Vargas-Arenas 1979, pp. 28, 206), and the Río Guapo component dated to 1630 ± 100 BP (AD 270; Y-1231) (Rouse and Cruxent 1963, p. 156). All of these are found on the eastern Venezuelan coast. These data may suggest that the pottery from Los Cumaneces was carried there by the avant-garde of the Middle Orinoco Ronquinan Saladoid, who might have migrated northwards through the eastern plains towards the east-central coast, rather than arriving at La Tortuga Island from the Venezuelan coast further east (see Rouse 1986). Fulvia Nieves (1992, p. 163) suggested that Arauquinoid people penetrated the Lake Valencia Basin from the Middle Orinoco plains and from there expanded to the Río Chico area. Antczak (1999) agreed that the first movements of Arauquinoid people to the north would have happened as proposed by Nieves, but suggested that once the Valencia style was developed, Arauquinoid influences penetrating the Lake Valencia Basin would have ceased. Nevertheless, the Río Chico area might have continued its relationship with the Middle Orinoco quite independently through the eastern plains. As long as both hypotheses lack the support of archaeological evidence, they pose challenges for future research. The interactions between the ancestral Arauquinoid and Valencioid populations might have taken forms and experienced dynamics different from those between the Arauquinoid and the Río Chico-area societies. These considerations gain strength when we assume the relational view of reality. This view leads us to consider that the Arauquinoid and Valencioid culture bearers and their neighbours figured not only as integral parts of the world of relations, and not only as interrelated with each other. Also true is that their very existence, identity and socio-cultural habits were constructed, maintained and transformed by their ongoing, fluid interactions with one another (Watts 2013). The nature and dynamics of these interactions have so far been left undefined due to the scarcity of adequate archaeological data and sound chronological indicators.

#### Temper

Lathrap (1970) argued for a strong correlation between ceramics and language. He proposed that groups affiliated with the Cariban linguistic family were the producers of pottery tempered with the spiculae of a freshwater sponge called *cauixí*. Tarble and Zucchi (1984) further proposed that the producers of *cauixí*-tempered pottery, specifically of Arauquinoid-Valloid affiliation, dominated the Middle Orinoco region during the second part of the first millennium AD. However, *cauixí*-tempered pottery was not just a Middle Orinocan trademark. Late pre-colonial and early colonial sites located in the western high savannas (*llanos*) of Venezuela, in the *llanos* of Colombia, and farther south as well, recurrently yielded spiculae (Gassón 2002, p. 263). In early colonial times these vast regions were considered mutually dependent (Morey 1976; Morey and Morey 1975). Taken together, they were conceived of as the Orinoco System of Regional Interdependence, based on peer polity exchange, and linked by the ethnohistorically known †*Achagua* Amerindians (Arvelo-Jiménez et al. 1989; Arvelo-Jiménez and Biord 1994). Though we share the main critique formulated by Zucchi and Gassón (2002) and Gassón (2007a) regarding the models of regional interdependence systems, we are confident that future research will provide a sounder archaeological basis to support the anthropological constructs of ‘peer-polity-exchange’ on a case-by-case basis.

The acceptance of *cauixí* as a distinctive feature of the cultural baggage of Cariban-speakers poses some problems. In the Middle Orinoco and in adjacent regions where high confluence and interaction of various ethnic/linguistic groups was reported in late pre-colonial and early colonial times, the use of *cauixí* temper might be expected not only among Cariban-speaking but several non-Cariban-speaking pottery-making groups as well. The identification of the Arauquinoid ceramic style with the historically known †*Otomaco* Amerindians from the Orinoco River region suggests that not all *cauixí*-tempered ceramics were necessarily related to Cariban-speaking groups (Gassón 2002, p. 295). It should be noted that the †*Otomaco* language pertained to the extinct Otomacoan linguistic family (Hammarström 2014, Table 3.1). A similar example comes from the Guarguapo Complex, or Barrancas Postclassic, which endured in the Lower Orinoco until at least the early seventeenth century and represented the final phase of Barrancoid ceramic development (Rouse and Cruxent 1963, p. 155; Sanoja 1979, pp. 277–280, Fig. 19). This complex was hypothetically related to the Arawakan linguistic family (Lathrap 1970, p. 127) and was characterized by the adoption of some typically Arauquinoid attributes including *cauixí* temper (Boomert 2000, p. 120). José Oliver (2016, pers. comm.) also observed that *cauixí* is not the sole tempering agent in all Arauquinoid ceramics. In his recent excavations in the Atures Middle/Upper Orinoco border region he found that sand was often mixed with *cauixí*, and that there were other combinations that involved *cauixí* as well as other tempers. It is to be expected that if the *cauixí* temper was not exclusive to Cariban-speakers’ pottery in the Middle Orinoco, it would also not distinguish it in the north. In fact, *cauixí* temper was reported in north-central Venezuela only sporadically. One example was given by Nieves (1992, p. 162), who reported the presence of *cauixí* in pottery linked to the Valencioid/Arauquinoid series at the Chupaquire site, in the Barlovento region located in the northeastern corner of the Valencioid Sphere of Interaction. Another example is a painted vessel of composite form tempered with *cauixí* that was reported in the Las Aves de Sotavento Archipelago. This vessel pertained to the Dabajuroid component, dated to between 690 ± 80 BP (AD 1260; I-16,286) and 470 ± 80 BP (AD 1480; I-17,218), and related to the Arawakan-speakers (Antczak and Antczak 2015b). Some *cauixí* temper was found on Trinidad, in the Bontour and St. Joseph Arauquinoid pottery complexes, but this may be considered as imported from the Lower Orinoco area (Boomert 1985, p. 107; Boomert et al. 2013, pp. 107–108, 140). In the Guianas, the pottery with Arauquinoid stylistic traits was mainly tempered with crushed sherds; *cauixí* temper is absent (van der Bel 2015, p. 585).

In the Orinoco River Basin, freshwater sponges (15 different species) are particularly abundant. The most common is *Drulia browni*, encountered in peculiar nests which are exposed during the drought cycle. Freshwater sponges are not abundant in north-central Venezuela, specifically not in the Lake Valencia Basin. In currently polluted Lake Valencia, only sponges of the genus *Ephydatia* have been reported. These still lack identification by species due to their lack of gemmules (Pauls and Volkmer-Ribeiro 1997). In the northern Tuy and Guaire rivers to the east, the presence of freshwater sponges is nil. Thus, we may argue that *cauixí* was an ecologically limited temper, replaced in the north by easily procured coarse sand, quartz and mica. In this case a relationship between the Cariban-speakers and *cauixí* temper is probably valid, but not exclusive, and should be examined on a case by case basis.

#### Slip

Slip is an important attribute to take into account in the comparative study of Valencioid and Arauquinoid pottery. This feature often appeared in the pottery of the Valencioid series, leading Wendell Bennett to go so far as to label it ‘the Valencia Phase trait’. Its origin was, however, attributed to a vaguely defined ‘external influence’ (Bennett 1937, p. 137). Antczak (2000) demonstrated that although the use of red slip is quite common in Valencioid pottery, in fact several different slips were in use, ranging in colour from light and dark red through red–brown and buff to white. Moreover, different slips were often applied to various zones of the same vessel or human figurine. The comparative study of the chemical composition of diverse slips, and the use of such slips on the various zones of anthropomorphic and zoomorphic figurines, *adornos*, effigy vessels and cooking/storage pots may yield new insights into Arauquinoid–Valencioid pottery resemblances (see Pino et al. 2013).

#### Firing Temperature

Using X-ray diffraction, Fournier and collaborators (Fournier et al. 2000; Fournier 2014) carried out a country-scale study on the mineralogy of pre-Hispanic ceramics from 24 speleological sites of Venezuela. They found that, in general, ceramics from the Middle Orinoco (caves and shelters of Amazonas and Bolívar States), as well as from Valencioid sites in north-central Venezuela (Cruxent and Guanasna caves and shelters, Miranda State), were fired using temperatures of less than 900 °C. These sites fall within the regions historically occupied by Cariban-speakers. Similarly, some ceramic materials from the caves of today’s Falcón State featured temperatures of less than 900 °C. However, in the same state, and contrasting with the rest of the nationwide sample, there was a region with a distinct single cluster of ceramics that reached temperatures of more than 900 °C. These localities are found in the peninsula of Chichiriviche, which clearly overlapped the historical Arawakan-speaking (†*Caquetío*) western region of Falcón State. The overlap is relatively close to the suggested historical border between the Arawakan and Cariban territories (Durbin 1977) (Fig. 4). Certainly, the reported differences are not fully conclusive for distinguishing pottery technologies within both Amerindian groups; moreover, the dating is limited and problematic. Nonetheless, this state of affairs now opens novel research possibilities for this topic.

### Landscape Engineering

The requisite knowledge and technological and social capability to build earthen structures may be considered additional traits perhaps serving to strengthen the link between Valencioid people and their Arauquinoid ancestors. Pre-colonial earthen structures have been reported in Venezuela since the nineteenth century (Marcano 1971a; Jahn 1927; Gassón 2002; Rey-González 2007). Systematic research into mounds or elevated platforms, raised cultivation fields and causeways in the western *llanos* of Barinas State began in the 1960s (Zucchi 1968, 1973; Spencer et al. 2014). Unfortunately, research into these phenomena in the densely populated and urbanized Lake Valencia region was long in coming. A century ago, the mounds were already being ‘destroyed in a hasty and unguided search for specimens’ (Spinden 1916, p. 326). Today the vast majority of these structures have already disappeared (Antczak and Antczak 2006).

Alberta Zucchi (1978) considered that the Barinas earthworks were related to the westward expansion of Arauquinoid *cauixí*-tempered pottery makers from the Middle Orinoco area. Materials of Arauquín style were found at various localities in the lower *llanos*, associated with probable mounds and also ceramics tempered with *cauixí* (Gassón 2002, p. 261). According to radiocarbon dating, from 950 BP (AD 1000) onwards, the Orinoco and Amazon Basins experienced the expansion of tropical forest groups of people, which reached its height in the Middle Orinoco with the aforementioned Arauquinoid expansion (Gassón 2002, p. 256). Due to this expansion, many smaller groups from the Middle Orinoco were already displaced to the west by 750 BP (AD 1200) (Zucchi 1978). In consequence, several archaeological complexes began to evolve in the western *llanos* using the raised-field cultivation system on the seasonally inundated plains (Zucchi and Denevan 1979). Further archaeological research on the western *llanos* focusing on how sociopolitical complexity and interactions might have been embodied in the spatial distribution and use of these earthworks is needed (Garson 1980; Spencer and Redmond 1992; Gassón 1998, 2002).

On the eastern shore of Lake Valencia, dozens of earth mounds were still visible at the end of the nineteenth century (Marcano 1971a), and between 22 and 26 in 1903 (Jahn 1932, 4). Further research revealed the presence of mounds on the western shores of the lake (von den Steinen 1904; Requena 1932; Osgood 1943; Bennett 1937; Peñalver 1965, 1967, 1971). However, the structures themselves were largely perceived as bounded containers of the most conspicuous material remains of the Valencia culture, rather than as integral elements of the cultural landscape in themselves (Antczak 1999). The mounds on the eastern shore of the lake had circular bases of 20–40 m in diameter and were 2.5 m in height. The largest one had an elongated form, measuring 130 m long, 63 m wide, and 3 m high (von den Steinen 1904, 104). Radiocarbon dates from the mounds on both lake shores show internal consistency, ranging from 1025±115 BP (AD 925 [Peñalver 1969]) to 1000±70 BP (AD 950 [Rouse and Cruxent 1963, 155]) (Table 1). While the Lake Valencia mounds await future systematic research, their very presence may cautiously be used to strengthen the claim for the Arauquinoid origin of the Valencioids.

**Table 1:** Radiocarbon, fluoride, and thermoluminescence dates (uncalibrated) from archaeological sites in north-central Venezuela associated with the Valencioid series

Site/excavation unit	Sample code/lab	Sample type/context/depth (cm)	Uncalibrated BP	AD	References
*North*-*central Venezuela mainland*					
Los Cerritos, eastern shore of Lake Valencia	Geochron Lab. Inc.	Human bones; 80 cm	1025 ± 115	925	Peñalver (1969)
La Mata, eastern shore of Lake Valencia	Y-630	Artificial earthen mound	1000 ± 70	920	Rouse and Cruxent (1963)
La Mata, eastern shore of Lake Valencia	Y-632	Artificial earthen mound	1000 ± 100	920	Rouse and Cruxent (1963)
La Mata, eastern shore of Lake Valencia	Y-631	Artificial earthen mound	980 ± 110	940	Rouse and Cruxent (1963)
Valle de Chuao, central-western Caribbean coast^a^	IVIC	Pottery (?)	744 ± 98	1206	Morales (1984)
Ricardo Zuloaga Cave	Geochron Lab. Inc.	Wooden stick	995 ± 75	955	Urbani (1998a, b)
Caracas, ‘Man of Caracas’^b^	IVIC-486	Human bones	1000–2000	0–1000	Tamers (1969) Anonymous (1970)
El Cafetal, Caracas Valley^c^	Teledyne Isotopes	Human bones	490 ± 75	1460	de Bellard-Pietri (1982)
*North*-*central Venezuela Caribbean islands*					
Cayo Sal Island, CS/D/1^c^	I-16,287	Hearth; 35 cm	750 ± 100	1200	Antczak and Antczak (2006)
Cayo Sal Island, CS/P6	Beta-209967	Shell^e^	1150 ± 60	1160–1390^d^	Antczak et al. (2008)
Cayo Sal Island, CS/P1	Beta-209968	Shell^e^	1070 ± 60	1270–1440^d^	Antczak et al. (2008)
Cayo Sal Island, CS/D/2	Beta-176597	Shell^e^	870 ± 60	1410–1500^d^	Antczak et al. (2008)
Dos Mosquises Island, DM/A/C/10^c^	I-15,087	Hearth; 45–47 cm	470 ± 80	1480	Antczak and Antczak (2006)
Dos Mosquises Island, A/B/9^c^	I-16,294	Hearth; 38 cm	490 ± 80	1460	Antczak and Antczak (2006)
Dos Mosquises Island, DM/A/C/11^c^	I-15,088	Hearth; 38 cm	520 ± 80	1430	Antczak and Antczak (2006)
Dos Mosquises Island, DM/A/1K^c^	I-16,279	Hearth; 43–49 cm	680 ± 80	1270	Antczak and Antczak (2006)
Dos Mosquises Island, DM/CN/1^c^	Beta-176599	Shell^e^	560 ± 60	1290–1440^d^	Antczak and Antczak (2006)
Dos Mosquises Island, DM/CN1B^c^	Beta-176599	Shell^e^	1120 ± 50	1200–1340^d^	Antczak and Antczak (2006)
La Pelona, PL/CN/4^c^	Beta-178239	Shell^e^	1150 ± 60	1160–1330^d^	Antczak et al. (2008)
La Pelona, PL/CN/3^c^	Beta-176600	Shell^e^	1070 ± 60	1230–1420^d^	Antczak et al. (2008)
La Pelona, PL/CN/1	Beta-176601	Shell^e^	870 ± 60	1390–1540^d^	Antczak et al. (2008)
Isla Larga, IL/A1^c^	Beta-206746	Shell^e^	1060 ± 60	1240–1420^d^	Antczak and Antczak (2006)
La Orchila Island, OR/F/A/6^c^	I-16,323	Hearth; 63 cm	580 ± 80	1370	Antczak and Antczak (1993)

All are radiocarbon dates unless otherwise indicated: ^a^ Thermoluminescence or ^b^ fluoride. ^c^ Samples taken from Valencioid deposits; the remaining island samples shown in the table come from shell midden layers without culturally attributable materials. ^d^ Dates cal to 2σ Sigma. ^e^ Only *Lobatus gigas* shells with circular human-made perforation in their spires (this means that the animals were ‘killed’ by humans) were used as samples. Note that the codes of excavation units used here are those stated in the laboratory references

### Chronology

We suggest that Cariban-speaking groups, from 1150 BP to 1050 BP (AD 800–900), migrated west, north and east from the Middle Orinoco following Villalón’s geographical features (Villalón 1987). This correlates well with the radiocarbon dates of the Valloid series at the Matajey site between 1455 ± 140 BP and 995 ± 160 BP (AD 495–1000) (Tarble and Zucchi 1984, p. 442, Table 2). Indeed, the presence of Cariban-speakers at this site overlaps with the dates of their possible expansion to the West between 1150 BP and 1050 BP (AD 800–900). We further consider that the rough 1150 BP (AD 800) date for the beginning of Cariban-speaking groups’ expansion northwards is consistent with radiocarbon dates ranging from 1025±115 BP (AD 925) to 1000±70 BP (AD 950) for Valencioid material associated with the Lake Valencia Basin (Table 1).

After settlement in the Lake Valencia region, northward expansion from the Middle Orinoco area would then likely have continued primarily eastward through the Tuy River depression and the geomorphological corridor of Aragua-Barlovento, continuing from there into the Caracas Valley through the submontane geomorphological corridor of Aragua-Los Teques-Caracas and the Barlovento Plains (*llanada barloventeña*). This movement could have been relatively fast, because for the period between 1040 ± 110 BP (AD 955, GX22246) and 995 ± 75 BP (AD 1000, GX22745) the radiocarbon dates are associated with possible tools used for hunting oilbirds (*Steatornis caripensis*) in the Ricardo Zuloaga Cave (Urbani 1998a, b, p. 8). Interestingly, oilbird (in Spanish *guácharo*) hunting is still carried out by the eastern Cariban-speaking *Chaima* from the Caripe Mountain Range [Galán 1981; see also von Humboldt [1995, pp. 100–107] for Amerindian use of oilbirds in eastern Venezuela in late colonial times] and by western Cariban-speaking *Yukpa* and *Jápreria* in the Perijá Mountain Range (Straka 1980; Urbani et al. 2003). Also the *Barí* (Chibchan-speakers) still hunt these birds in the Perijá Mountain Range (Viloria et al. 1989). The Ricardo Zuloaga Cave is situated very close to sites that yielded ceramic material apparently linked to the Lira Cave, which featured the Valencioid El Pinar style reported by Cruxent and Rouse (1958, p. 322). The Ricardo Zuloaga Cave is also situated near La Botija Cave, which exhibited the Valencioid Las Minas style (Cruxent 1959a, b), as well as near Peñon de Guanasna, which revealed additional likely Valencioid materials from rock shelters and caves (Urbani 2000b, 2009). Within the Valencioid series, Cruxent and Rouse (1958) related the El Pinar style with the final dates of Period III of this series (1050 BP [AD 900]), in accordance with the presence of vertical strap handles and incised semi-circular appendixes. They also relate this style to Period V (AD 1500) due to its relationship with the El Topo style. In this context, we should also mention the age ascribed to the so-called ‘Man of Caracas’: between 1000 and 2000 BP (50 BC and AD 950) (Tamers 1969) (Table 1). In the Caracas Valley, the radiocarbon date of AD 1460 has been associated with an archaeological site located in the purported Cariban-speakers’ occupation area (de Bellard-Pietri 1982; Table 1). There also exist a series of post-AD 1200 dates for clearly Valencioid materials recovered on the islands of Los Roques and La Orchila (Antczak and Antczak 2006).

Late pre-colonial archaeological remains were also reported in the surroundings of the city of Caracas, specifically in Altos Mirandinos, Tacagua, Turgua, El Topo, and El Camino de Los Españoles (Marcano 1890; Cruxent 1946, 1951; Dupouy and Cruxent 1946a; Dupouy and Cruxent 1946b; La Salle 1952; Anonymous 1958; Rheinheimer-Key 1986; Gómez-Aular 1995; Rivas 1994, 1997; Amodio et al. 1997). Cruxent and Requena (1946) found a site with a large number of milling stones (*metates*) that overlapped the location of today’s Ciudad Caribia. Francisco de Paula-Alamo (1911, pp. 298–301) reported that abundant pottery, lithics, and artefacts made of bone and queen conch (*Lobatus gigas*), as well as beads and vessels with human remains inside, were found at the Peñon de Guaicaipuro and San Corniel sites in the Los Teques area. In the area of the Mamo River, west of Caracas, Amerindian remains were reported by Marcano (1971a). By the late nineteenth century, Marcano (1971a) provided an unusual observation on the elusive nature of the archaeological record in mountainous tropical forest in northern South America:

West and some four leagues from Caracas, along the margins of Mamo [River], there is a mountainous land that was cleared by 1820 in order to plant coffee. The earliest proprietors used to have the conviction that those forests were pristine. However, once they inhabited this area, they found numerous remains of primitive homes: perfectly made paths, stone weapons, pottery, urns, and utensils for the home […]. The creeks that descend from the top on the mountains were used to water the plantations by using trenches made once the terrain was leveled. The current proprietors preserved those irrigation channels that even today are active […]. Other neighbouring forests that were cleared in 1835 and 1841 also hid indigenous remains. (Marcano 1971a, p. 101)

Recently, Franz Scaramelli (2012, pers. comm.) found pre-Hispanic potsherds in Altos de Pipe, near Caracas. Arvelo et al. (2013) also reported pre-Hispanic remains along the Cuira River of the Cordillera de la Costa mountain range, 80 km southeast of Caracas. They comprise lithic materials (stone grinders, milling stones, stone chips, and possible sharpener stones), as well as pottery sherds of the Valencioid series ascribed to a chronological range of AD 1100–1600. The authors classified these sherds as similar to the pottery of the Topo Style of the Valencioid series (Cruxent and Rouse 1958; Herrera-Malatesta 2009, 2011), and the archaeological material collected by Nieves (1992) in the north-central coastal sites of Chupaquire and Cúpira. Two sherds were identified as part of the Memoid series (Arvelo et al. 2013). Rodríguez-Yilo (1999, p. 278; see also Amaiz 2000) described Memoid series remains at a lowland site in Guaribe (Guárico-Miranda State border), 50 km southeast of the Cuira River basin, dated to between 450 ± 50 and 400 ± 40 BP (AD 1500 and 1550, Beta 123290 and 123291). In the Cuira River basin itself, Arvelo et al. (2013) also located three pre-Hispanic sites under forest cover. These sites were situated over 500 masl along the lateral sides of the mountains (>40° inclination) and relatively close to their summits.

**Fig. 2:**
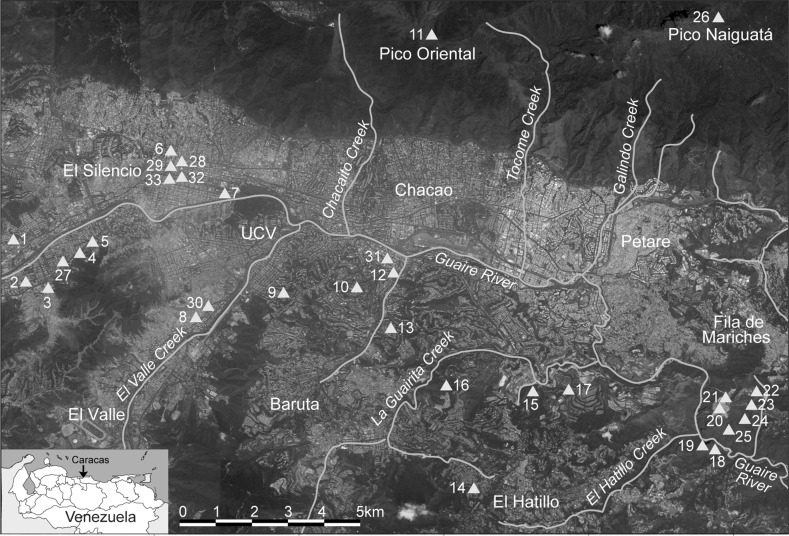
Locations of the late pre-colonial archaeological sites in the valley of Caracas city (the locations are approximate and do not include sites with petroglyphs). *Numbers* in this figure correspond to the numbers of the first column of Table 2. Base map, Dirección de Cartografía Nacional 1.100.000, sheet 6847. Base image, Google Earth™ © 2015 CNES/Astrium

**Fig. 3:**
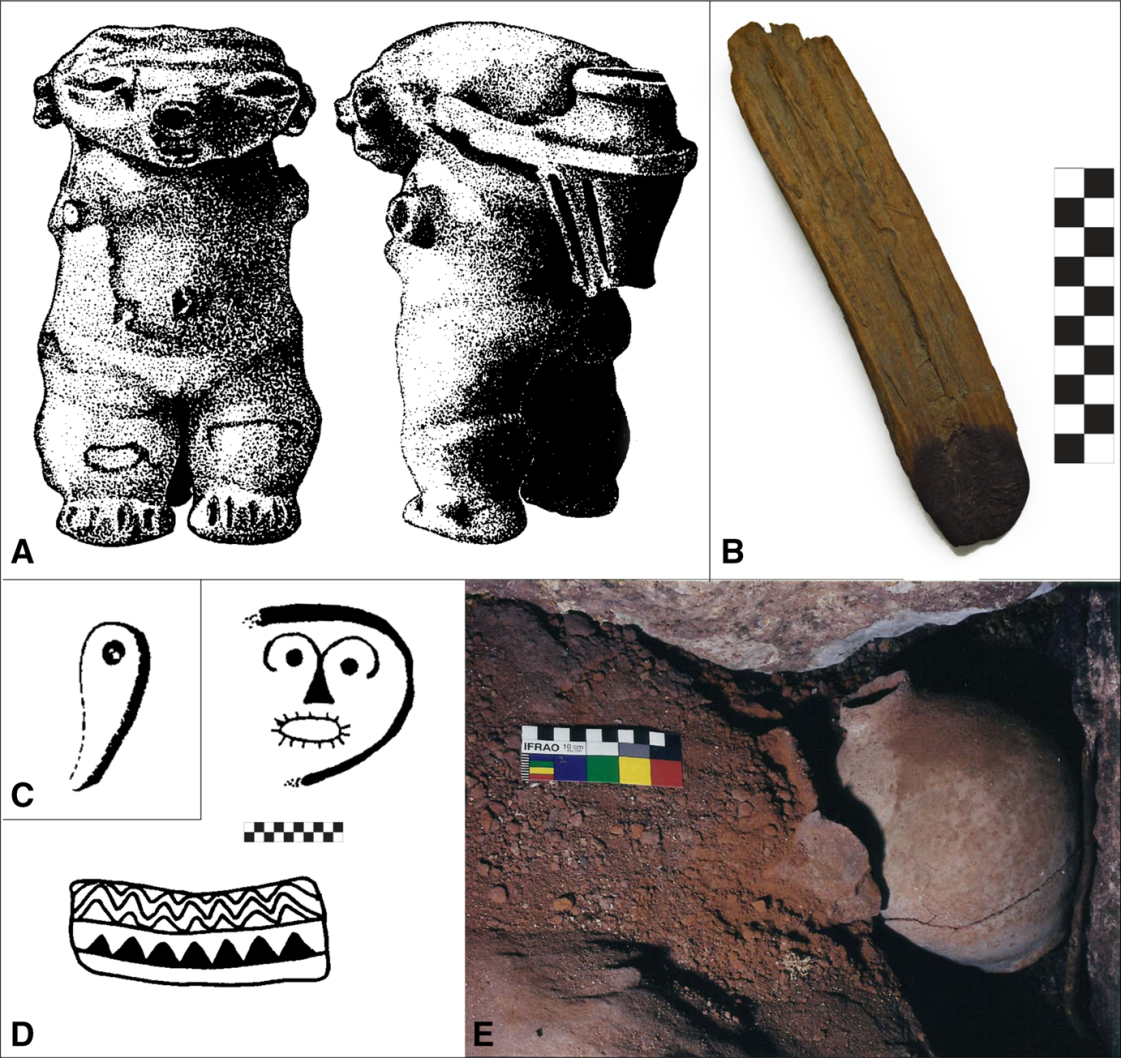
**a** Anthropomorphic figurine found near Guaire River in Fila de Mariches (Cruxent 1964; Table 2, #20); **b** possible torch from Ricardo Zuloaga Cave (Urbani 1998a, b; Urbani 2000b, 2009; Table 2, #18); **c** ‘tooth’ from the necklace of queen conch found at El Valle (Ernst 1872; Table 2, #8); **d** rock paintings from Los Carraos/Las Guacas Cave (Urbani 2000b, 2009); **e** *in situ* complete vessel from Peñón de Guanasna (Urbani 2000b, 2009; Table 2, #24) (graphic scale: 1 square = 1 × 1 cm). Images reproduced with permission from the Centro de Antropología, Instituto Venezolano de Investigaciones Científicas and the Departamento de Antropoespeleología, Sociedad Venezolana de Espeleología

Table 2; Figs. 2 and 3 summarize information about material culture, especially portable objects, recovered from the Caracas region. In addition, a large natural shelter with aligned rocks and petroglyphs in the Cordillera de la Costa mountain range was reported by Ernst (1956). Also, an anthropogenic accumulation of rocks was found in a flat area surrounded by rock shelters and caves with likely Valencioid material at Peñón de Guanasna (Urbani 2000b, 2009). Petroglyphs, moreover, were recorded in the Caracas Valley and its surroundings (Cruxent 1951; Jam 1955; Albornoz 1983; de Valencia and Sujo-Volsky 1987; Urbani 1998a, b) as well as rock paintings (Urbani 2000b, 2009). Using early historical documents, Biord (2007) suggested that between AD 1550 and 1600 in north-central Venezuela, indigenous peoples seem to have preferred lowland localities for the formation of dispersed or clumped multi-family settlements, as well as for temporary settlements. Furthermore, documented oral history in present-day southeastern Caracas mentions sites recalled as inhabited by Amerindians (Urbani 2000a, b, 2009). Finally, some elements of indigenous technology and material culture are reported to persist in contemporary Caracas (Quintero 1967).

**Table 2:** Late pre-colonial archaeological localities and findings in the valley of Caracas city

#	Site	Material	References
1	La Yaguara	Pottery associated with the ‘societies of Arawakan-speaking potters and agriculturalists’	Rivas (1997)
2	Montalbán	In 1968, a human skull was found; the remains were called ‘The Man from Caracas’ and dated to between 1000 and 2000 BP (IVIC-486)	Tamers (1969), Anonymous (1970)
3	Hacienda La Vega	J. M. Cruxent and A. Singer found some pottery similar to the potsherds found in the Parque Central area	Villasana (1972)
4	La Vega	In 1888, Vicente Marcano reported two skulls from an altered cemetery and some human remains found inside ceramic vessels	Marcano (1971a, b)
5	El Pinar	This is the site that gave its name to the El Pinar ceramic style of the Valencioid series	Cruxent and Rouse (1958)
6	Caracas city center	Mario Sanoja indicated at a conference held on June 16th, 2000 at the Museum of Natural Sciences of Caracas that some pre-colonial remains were recovered in the excavations carried out in the central part of Caracas	M. Sanoja (2000: Conference presentation)
7	Parque Central	A. Singer found Amerindian potsherds and carbonized particles in an excavation carried out during the second phase of the construction of the Central Park Buildings (Caldera, 1972). J. M. Cruxent identified the material as pertaining to the Valencioid series. Near Parque Central, Anonymous (1967) also reported ceramic vessels, but it was not specified if they were indigenous	Singer (2000, pers. comm.)
8	El Valle	Adolf Ernst reported an Amerindian cemetery from which he obtained ‘thirty objects in form of tooth’, possibly part of a necklace probably made from queen conch shell (*Lobatus gigas*)	Ernst (1872)
9	Santa Mónica	One funerary ceramic vessel was found with human bones inside	Navarrete et al. (1995)
10	Baruta	In 1888, Marcano reported carved ‘silex’	Marcano (1971a, b)
11	Las Mercedes - Valle Arriba	Reported presence of Amerindian ceramic material. Sherds from Calle Herrera Toro of Las Mercedes-Valle Arriba found by P. Jam	Jam (1958), La Salle (2005)
12	Silla de Caracas - Pico Oriental (El Ávila Nacional Park).	A. Jaimes reported pottery pertaining to the Valencioid series at the Silla de Caracas. A. Ernst indicated an unknown number of figurines (probably not more than five specimens)	A. Jaimes (1990, pers. comm. to F. Urbani); Jahn (1932)
13	El Cafetal (Colinas de Tamanaco)	Human bones (AD 1460) as well as an alleged pre-Hispanic gold object are reported	de Bellard Pietri (1977, 1982)
14	La Peñonera	J. M. Cruxent reported pottery similar to that recovered at Topo de Tacagua	Cruxent (1951)
15	La Lagunita	Reported carved ‘silex’	Marcano (1971a, b)
16	La Guairita (La Guairita Recreational Park)	Pre-colonial site with pottery	IPC (1997)
17	Iglesitas Cave	R. Hernández (2000, pers. comm.) found a skull and a canine tooth that J. M. Cruxent identified as pre-colonial	Urbani (2000a)
18	Ricardo Zuloaga Cave	Reported the finding of a wooden stick possibly for hunting of oilbirds dated to 995 ± 75 BP (AD 1000), as well as a torch	Urbani (1998a, b, 2000b)
19	Cave of the Carraos or Las Guacas	Reported poles for oilbird hunting apparently associated with Amerindian pictographs	Urbani (2000b)
20	Cave of the Figulina (southeast of Petare district)	J. M. Cruxent reported an anthropomorphic figurine near the Guaire River, Fila de Mariches	Cruxent (1964)
21	Gran Abrigo del Peñón de Lira	R. Hernández (2000, pers. comm.) reported many potsherds being found. Possibly this is the same site visited in 1948 by J. M. Cruxent and Colonel B. R. Lewis	Hernández (2000, pers. comm.), Cruxent and Rouse (1958), Urbani (2000a)
22	Lira Cave	Reported pottery related to El Pinar style	Cruxent and Rouse (1958)
23	Requena Cave	R. Hernández (2000, pers. comm.) reported apparent human remains (femur, skull and tooth). This cave has been destroyed by a quarry	Urbani (2000a)
24	Shelters and caves of the Peñón de Guanasna	Urbani (2000b, 2009) reported 11 speleological sites with likely Valencioid pottery. See also Barroeta Lara (1957)	Barroeta Lara (1957), Urbani (2000b)
25	La Botija Cave	Reported ceramic vessel classified as pertaining to the Valencioid series	Cruxent (1959a), Cruxent (1959b)
26	Pico Naiguatá (El Ávila Nacional Park)	Potsherds, some of them red-slipped	Antczak and Antczak (2006)
27	El Paraíso	One female figurine with canoe-shaped head	Cruxent and Rouse (1958, vol. 1: 323), Requena (1947: Figs. 18–21)
28	Asamblea Nacional (National Congress Building)	Spanish Maiolica associated with a Valencioid sherd	González-Jukisz (2015, pers. comm.)
29	Escuela de Música José Ángel Lamas	Trapezoid nephrite axe and jade pendant	Sanoja et al. (1998)
30	El Valle-Garzón	Axe found by Beld-Marstio in 1947	La Salle (1953)
31	Valle Arriba	Conch and sherds found by Mathias-Brewer (1952) and Ruz-Brewer (1953)	La Salle (1953)
32	Downtown Caracas	Cylindrical clay body stamps (*pintaderas*), lithic axes, and an oval jade pendant	Sanoja and Vargas-Arenas (2002)
33	Chancellery Building	Presence of apparent Valencioid sherds	Linero-Baroni (2008)

The numbers in the first column correspond to the numbers in Fig. 2 (see also Fig. 3)

**Fig. 4:**
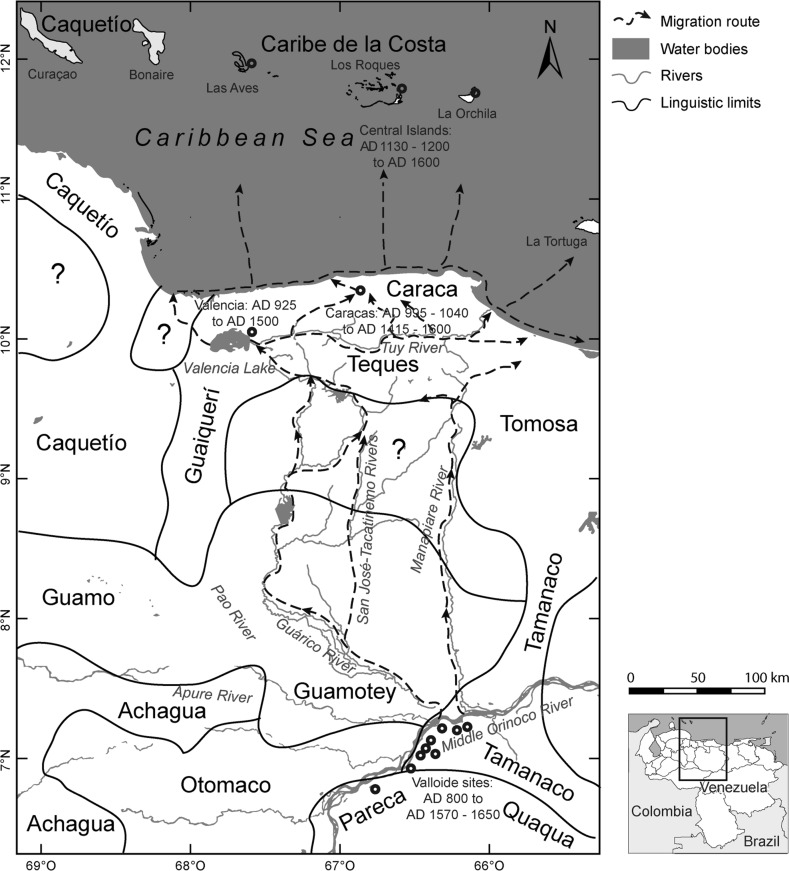
Suggested migratory routes of the Carib-speakers with respect to the distribution of the indigenous languages of the Venezuelan macro-region at the time of European contact (redrawn after Durbin 1977)

The above-discussed archaeological data and their temporal frame coincide with the spatial distribution of the Coastal Cariban-speakers as proposed by linguists (Fig. 4), as well as with the distribution of historically known indigenous groups in north-central Venezuela at the time of the Contact, specifically the †*Teques*, †*Caraca*, †*Mariches*, and †*Meregotos* (Biord 1992, 2001; Antczak 1999; Rivas 2001). These groups shared the same temporal position of historically-known Middle Orinocan Cariban-speakers, for example the †*Pareca*, †*Otomaco*, and †*Tamanaco* (Durbin 1977; Fig. 4). These Cariban groups, as evidenced by the timing indicated in Table 1, experienced apparently fairly rapid spatial dispersion. The Coastal Cariban-speaking groups seem to have been hegemonic in north-central Venezuela until well into the period of European contact, and the Cariban language might have been used as the *lingua franca* within the Valencioid Sphere of Interaction. Sanoja et al. (1998) excavated a jade pendant and trapezoid nephrite axe in Caracas (year of foundation AD 1567) similar to axes found in Los Roques Archipelago and Nueva Cádiz (Antczak and Antczak 2006). These axes cohere with the suggested chronology of the late Valencia tradition during the Contact period (Antczak and Antczak 2015). In addition, Sanoja and Vargas-Arenas (2002) excavated the earliest Caracas church, San Sebastián. Historical records indicate that it was founded above the camp where the Spanish conqueror Diego de Losada settled in 1567. Here, Sanoja and Vargas-Arenas (2002) found a *caney*-like structure incorporating a domestic context with indigenous materials, including lithic axes, cylindrical clay body stamps (*pintaderas*) and an oval jade pendant. Amerindian materials were associated with Spanish *Columbia Plain* majolica dated to the 1580s (Sanoja and Vargas-Arenas 2002). These authors also reviewed the earliest official Caracas Council documents, finding that between 1574 and 1579, the ceiling structure of this church was made of palm leaves, a local Amerindian resource. In addition, a likely late Valencioid sherd associated with Spanish majolica was found beneath today’s Venezuelan National Congress building in downtown Caracas (N. González-Jukisz 2015, pers. comm.). Beyond this region, Valencioid potsherds were reported associated with Spanish earthenware in the early Spanish town of Nueva Cádiz (year of foundation AD 1528) on the Island of Cubagua (which had featured previous temporary Spanish settlements since the first decade of the sixteenth century) in northeastern Venezuela (Cruxent and Rouse 1958 vol. 2, p. 318; Antczak and Antczak 2015a). Other possibly Valencioid potsherds were reported from La Blanquilla Island and associated with European pottery (Antczak and Antczak 1991). Fragments of Valencioid pottery figurines were also found on Margarita Island (Antczak and Antczak 2015a). In the Middle Orinoco area, dates associated with Valloid-series materials are recorded until the mid seventeenth century, indicating the clear presence of archaeologically defined Cariban-speakers in early colonial times (Tarble and Zucchi 1984). Archaeological evaluation of interactions between inhabitants of the Middle Orinoco and north-central Venezuela during colonial times still awaits systematic research (Antczak 1999).

## An Alternative Migration Route and Its Timing

The fluvial-riverine dispersion route hypothesis, which has nested for more than half a century in Venezuelan archaeology, has limitations imposed by the temporalities and topographies of socio-cultural landscapes. With the passage of time landscapes became increasingly populated, and the sociopolitical and linguistic entities inscribed tangible and intangible boundaries in them (see Løvschal 2014). Such boundaries unfolded in constant flux and transformation, making the routes of mobility and exchange dependent on fluctuating sociopolitical interrelations and interdependencies. Enemy territory avoided today would turn into friendly space tomorrow. Thus, while discussing the possible migratory route of the Orinocans, we adopted a macroregional diachronic perspective drawing from archaeology and ethnohistory. Furthermore, the topography across the South American Lowlands is far from uniform. In the geographical borderlands, where the lowlands approach the highlands, navigation upstream becomes difficult if not impossible. These environmental constraints may pose serious challenges to the notion of effective fluvial-riverine mobility evolving over many generations of lowlanders.

**Fig. 5:**
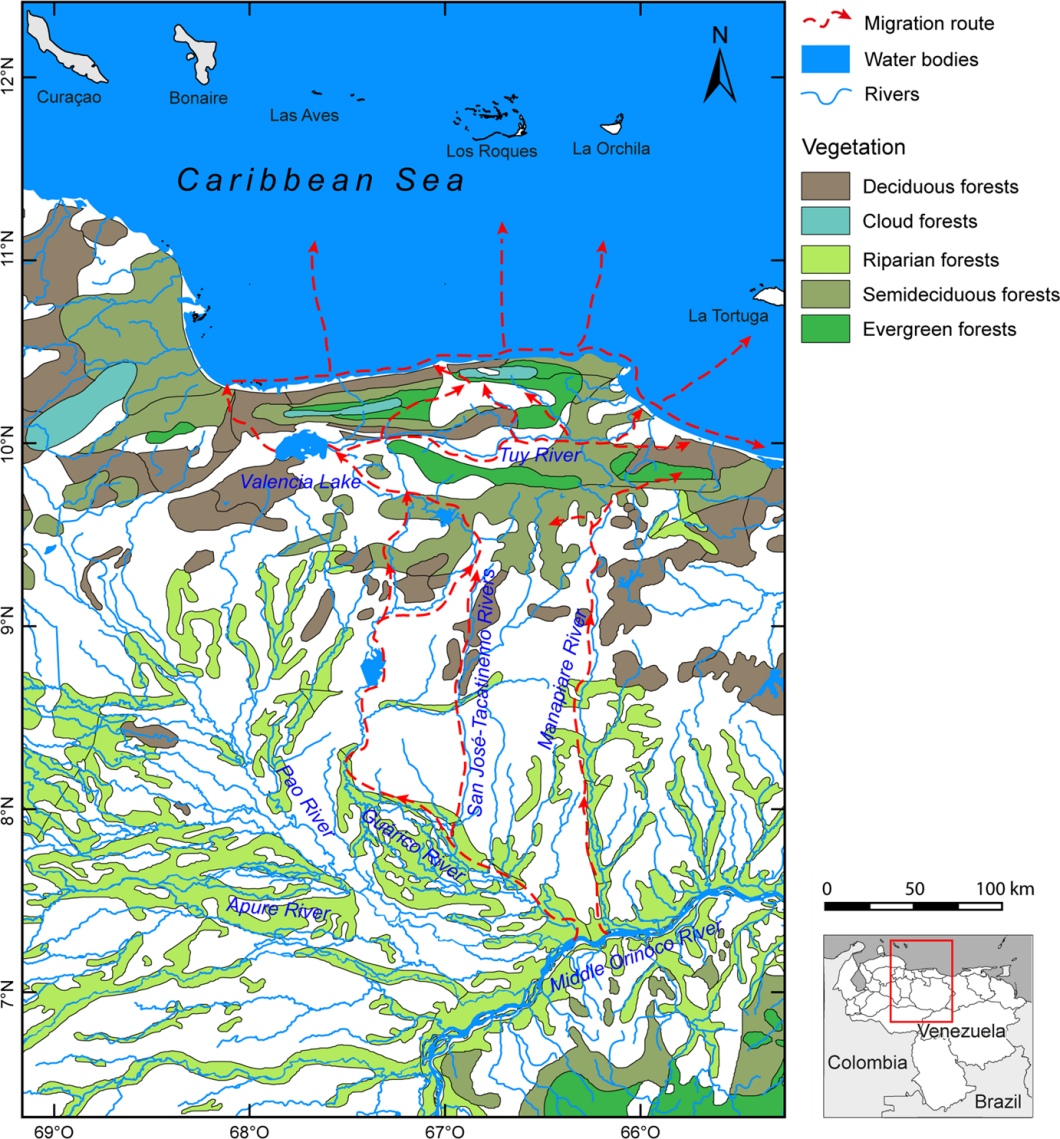
Suggested migratory routes of the Carib-speakers with respect to ecological zones of the macro-region (redrawn after Huber and Alarcón 1988)

Taking the above into consideration and pondering the available radiocarbon dates, we suggest that Cariban-speaking groups from the south could have entered north-central Venezuela through the La Puerta-Villa del Cura-Cagua geomorphologic corridor or *abra* (Figs. 1, 3). This corridor opens northward precisely in the area of the archaeological site of Los Cerritos on Lake Valencia’s eastern shore, dated to 1025±115 BP (AD 925) (Table 1). Penetration through this corridor probably first occurred without either permanent occupation or direct entry into the central region, and without passing through the mountainous Serranía del Interior characterized by humid evergreen rainforest and cloud forest (Huber and Alarcón 1988). In principle, this difficult-to-breach mountainous area would not have been the least cost path (c.f. Bell and Lock 2000). Paraphrasing Barbara Bender (2001, p. 75), it must have been rather an almost entirely unfamiliar landscape of movement for the Amerindians from the Orinoco region (Figs. 1, 5). Even today, crossing this intricate mountainous area is difficult: experienced local hunters report that it requires several effortful days (F. Urbani 2014, pers. comm.). In fact, until the 1970s, no proper roads existed through these mountains due to the technical difficulties and cost of such construction (F. Urbani 2014, pers. comm.). Nevertheless our suggestion does not exclude the likely presence of intermountain ‘Indian paths’ (in Spanish *caminos de indios*). For example, Marcano (1971a) mentioned that in north-central Venezuela there existed ‘precolumbian paths’ between Carayaca and Tuy, and ‘Indian paths’ between the Tuy River and the Caribbean coast of Chuspa. In any case, all the effort of crossing this mountain range might have been worthwhile to avoid potentially hostile nearby territories inhabited by Arawakan-speakers such as the †*Caquetío* to the west (Fig. 4; see also Muysken and O’Connor 2014, map 1.1. for a more general panorama). We also note that this mountain range—as opposed to more open surrounding areas—might have hindered the preservation and discovery of possible archaeological evidence confirming a crossing or crossings on the part of Cariban-speakers (see above Marcano 1971a, Arvelo et al. 2013).

Indeed, environmental factors might have proved important in both constraining and enabling the expansion of Cariban-speakers into the north-central region. According to the archaeological record, the spatial distribution of Valloid sites seems to suggest a close relationship with river networks (Tarble and Zucchi 1984). Based on this evidence, we suggest that northward expansion primarily followed the Guárico, San José/Tacatinemo and Manapire Rivers of the Venezuelan central *llanos*, and that these possibly constituted the main migration routes overall (Fig. 1). The Pao River and its connection with the Paíto River is the only other possible waterway (already suggested by Cruxent and Rouse 1958). However, we consider that this latter route was likely less frequented by the Cariban-speakers given possible competition and violent interactions with Arawakan-speaking groups settled in this area (Fig. 4). Both these routes offered navigable conduits from the Middle Orinoco and ended a relatively short distance from Lake Valencia, in the vicinity of the present-day town of San Juan de Los Morros, Guárico State. The Cariban-speakers would have entered the Lake Valencia Basin through the corridor La Puerta-Villa del Cura-Cagua, then proceeded east along the Tuy River and through the Caracas Valley. Before the entry to the Lake Valencia Basin, the environment is dominated by savannas associated with deciduous, semideciduous, and riparian forests (Fig. 5). These are very similar to those of the Orinoco area (Huber and Alarcón 1988). The savannas also included the typical matrix of savanna-*Curatella americana* (Dilleniaceae, in Spanish *chaparro*) shared by the Middle Orinoco region and the far north of the Orinoco River up to ~100 km into the central *llanos* (B. Urbani, pers. obs.). This setting closely resembles environments historically occupied by the Cariban-speaking †*Pareca* and †*Tamanaco* of the Middle Orinoco. Paleobiolinguistics may contribute to a better understanding of the biodiversity known to the Arauquinoid and the Valencioid peoples in this area (see, for example, Brown 2010; Brown et al. 2016).

We further argue that the expansion northwards must have commenced at least by 1150 BP (AD 800), since by 1050 BP (AD 900) Cariban-speaking makers of Valencioid pottery were present in north-central Venezuela. By this time, their pottery had already acquired a series of distinctive decorative traits. The new lands around Lake Valencia and the adjacent Caribbean coast were not a *terra nullis* (sensu Gosden 2004) for the newcomers. Even if in the early stages of the migratory route they were able to avoid encounters with Arawakan-speakers, they still had to interact with them in the Lake Valencia Basin. These early interactions were characterized by archaeologically as yet undisclosed processes of fusion, fission, and friction, or some combination of these three (Rivas 2001). Most probably, friction and violent skirmishes occurred in the Lake Valencia surroundings as the Barrancoid had to cede their settlements to the newcomers and move to the Caribbean coast. However, the Ocumaroid and Barrancoid inhabitants already on the coast seemingly entered into a long phase of neighbourly, rather peaceful interactions with the Lake Valencia Basin Valencioid culture. These relations lasted probably until the sixteenth century (Rivas 1997; Antczak and Antczak 2006; Sýkora 2006).

The specific forms of interaction and the building of sociopolitical boundaries between the groups that would correspond to the specific pottery styles within the Valencioid series also await future research (see Antczak 1999; Rivas 2001). It seems possible that certain ‘peripheral’ Valencioid groups were located to the east of the Lake Valencia Basin settlements where Valencia-style pottery was found. These adjacent groups specialized in the production and exchange of an ‘archaeologically elusive’—that is, highly perishable—material culture, including feather adornments, wooden objects, waxes and dyes, textiles, hammocks, and basketry. The last manufacture is widespread among contemporary Cariban-speaking groups from Orinoquia such as the *Ye’kuana* and *E’ñepa*. However, at the same time, these groups produce very little decorated pottery (Henley and Matteí-Müller 1978; Rivas 1998). Similar elusive production might be hidden behind the ‘peripheral’ styles of the Valencioid series such as El Pinar, El Topo, and Las Minas (Rouse and Cruxent 1963).

It should be noted that only a part of the indigenous materials from north-central Venezuela comes from controlled archaeological excavations, while thousands of objects with little or no provenance data are held in private and public collections dispersed across the Americas and Europe (Antczak and Antczak 2006; Díaz Peña 2006; Guzzo Falci et al. 2017). In Venezuela there are large collections that remain to be re-contextualized. For example, an unstudied collection of lithic materials from the valley of Caracas, collected during the first half of the twentieth century by Luis R. Oramas (1884–1967), is held at the Museum of Sciences of Caracas (H. Moreno 2016, pers. comm.; B. Urbani, pers. obs.). These objects were collected in various localities: Chacao, Dos Caminos, La Trinchera-Caracas, Tacagua, Catia, Fila de los Mariches, Las Mayas and Conejo Blanco (El Valle), Las Adjuntas and La Majada (Macanao), Carapa and Carapita (Antímano), Cerro de Antímano, Caricuao, Sabana Grande, Mariches, La Vega, Barrancas (La Vega), Anauco Arriba-San José, La Guairita, El Pedregal and La Carlota (Chacao), and in the estates (*haciendas*) *Coche*, *Santo Domingo* and *Sosa* (El Valle), *Arvelo* and *La Cañada* (Petare), *Sabana Grande* (Bello Monte), and in the estate *Anzola*.

To the west of the north-central Arawakan-speaking Venezuelan territories which were purportedly invaded by the Cariban-speaking Valencioids, in the present-day states of Falcón and Lara, Oliver (1989) indicated the presence of an Arawakan ‘pre-*Caquetío*’ formation framed within the so-called Macro-Dabajuroid tradition. This formation could have given rise to the Tierroid and Dabajuroid traditions about 1250–1150 BP (AD 700–800) (note the parallelism of suggested dates for the beginning of the expansion of Cariban-speaking groups from the south). Dabajuroid pottery makers migrated during this period into the Yaracuy Valley and by 1050 BP (AD 900) had disseminated beyond it. In Yaracuy, these Arawakan-speakers entered into contact with groups from the macro-Cariban trunk who were the possible ancestors of the historic †*Jirahara* (Kaufman 1990). The former also occupied the Caribbean coast north of the Yaracuy depression and, further, the islands of Aruba, Bonaire and Curaçao. There is archaeological evidence of territorial control interactions that may suggest Arawakan-Cariban tensions. Between 750 BP (AD 1200) and the Contact Period, Arawakan (Dabajuroid) and Cariban (Valencioid) descent groups found themselves in dispute over access to and control of the central oceanic archipelagos of Las Aves de Sotavento, Las Aves de Barlovento and Los Roques (Antczak and Antczak 2006). In general, the presence of the Dabajuroid people in the northern part of present-day Falcón State seems to have restrained Valencioid settlement along the western coast. Valencioid ceramic traits are virtually absent in the ceramic assemblages recovered in the Yaracuy River depression as well as in the Quibor area (Wagner and Arvelo 1991; Arvelo and Wagner 1993; Arvelo 1995). These areas overlay the region occupied by early-Contact Arawakan-speakers, the †*Caquetío* (Fig. 4). However, new evidence and even some recently re-investigated sites on the coast of Falcón State demonstrate a more complex picture of human interactions. Not all archaeological traits recovered at these sites are Dabajuroid, posing a challenge to our understanding of the protohistoric Caquetío Arawakan-speakers (José Oliver 2016, pers. comm.). Also, in a preliminary archaeological study, Gómez and Gómez (1995) reported a mixture of Arauquinoid, Osoid, Tocuyanoid, Tierroid, and Valencioid pottery in La Cajara (Cojedes state), a site located between the central and western *llanos*, and northwestern and north-central Venezuela. Clearly, the spatial boundaries between Cariban, Arawakan and other indigenous speakers should not be considered impermeable.

Recently, Acevedo et al. (2017) have suggested the existence of a so-called pre-Hispanic ‘route of the variscite’ between the Los Roques Archipelago in Venezuela and northern Colombia. Using X-ray diffraction and electron probe micro-analysis, as well as spectrometry and petrographic studies, these authors suggest that the variscite beads (*cuentas*) of the Colombian Nahuange (AD 100–1000) and Tayrona (AD 1000–1600) cultures are made from variscite obtained from the Los Roques source on Gran Roque Island. Considering this fact, by AD 1200, raw material from the Los Roques quarry located in the Cariban-speakers’ (Valencioid) sphere of interaction must have crossed Arawakan-speakers’ domains in order to reach the Chibchan territories of the Tayrona to the west. Judging by the above-mentioned dates, this trade had to have begun before the occupation of Los Roques by the Ocumaroid and Valencioid (pre- AD 1000). However, archaeology thus far does not indicate human presence in Los Roques before AD 1000 and even afterwards there is no direct evidence of the indigenous use of the Los Roques variscite quarry (Antczak et al. 2017a). Thus, an increasing number of cases provide a puzzling diversity of archaeological materials that remain to be interpreted in terms of specific intergroup interactions, including mobility and exchange (Antczak and Antczak 2015b).

Dabajuroid and Valencioid stylistically diagnostic pottery has been recovered far from the homelands of its production and use and has been associated with fishing and scouting expeditions, exchange and trade missions, warfare and cooperation (Cruxent and Rouse 1958; Rivas 2001; Antczak and Antczak 2015b). In fact, pottery associated with purported Arawakan-speakers was reported dispersed along the Southeastern Caribbean coast and islands. For example, Tocuyanoid pottery was reported in Ocumare Bay (Sýkora 2006) and at the Cerro Machado sites (Rouse and Cruxent 1963). Ocumaroid ceramics are concentrated in the coastal bays of the central-western Caribbean coast between Choroní and Cepe (Rouse and Cruxent 1963; Morales 1984; Álvarez and Casella 1983; Martín 1995). Dabajuroid pottery has been found in the Los Roques Archipelago (Antczak 2000); on La Tortuga Island (Antczak 1999); at the Campoma site (Wagner 1972); and on Margarita Island to the east (Cruxent and Rouse 1958). Valencioid potsherds were detected in north-central Venezuela (Antczak and Antczak 2006; Herrera-Malatesta 2011); on the central-eastern coast (Nieves 1979, 1983, 1992); and far to the east on the islands of Cubagua and La Blanquilla, while fragments of Valencioid figurines were found on Margarita Island (Cruxent and Rouse 1958; Antczak and Antczak 1991, 2015a). All these sites contain a perplexing multiplicity of archaeological materials repeatedly awaiting explanation in terms of intergroup interactions and mobility. Finally, it is noteworthy that today’s Orinoquian Cariban-speaking *Ye’kuana* are known as notable canoeing people (Coppens 1971, 1981). In October 2016, a *Ye’kuana* canoe propelled by non-indigenous paddlers successfully crossed 135 km between the central Venezuelan coast and the Los Roques Archipelago in 29 h, re-enacting late pre-colonial Valencioid voyages (Caribe 2016).

## Re-focusing on Archaeolinguistics

References to language have frequently appeared in previous sections of this paper. Using the direct historical approach, we repeatedly crisscrossed the Columbian threshold to suggest a possible interrelation between early colonial sociocultural plus linguistic evidence and late pre-colonial archaeologically-exposed phenomena. In this section, we address an example of interrelationship between archaeological material culture and Cariban languages. This example could serve to advance archaeolinguistics in the study region.

Durbin (1985) found certain affinities between extinct Cariban languages from the east-central coast and the series of dialects in use by *Yukpa* and *Jápreria* Amerindians who nowadays inhabit the Sierra de Perijá mountain range on the Venezuelan–Colombian border. Going further, he suggested that the extinct †*Opone* and †*Carare* Cariban languages of northeastern Colombia were more closely related to the *Yukpa* dialect series than to any surviving Cariban language. This distribution suggested to Durbin a movement of Cariban-speakers from the eastern and central Venezuelan coast through the plains into the Lake Maracaibo area, then northward into the Sierra de Perijá and also southward through the foothills of this Sierra, finally following the course of the Magdalena River (Durbin 1977, p. 30, 1985, pp. 346, 349). Oliver (1990, p. 89) suggested that the displacement of the Cariban-speakers from east to west could have originated at a more ‘centric’ point on the inland plains (*llanos*). From this point, the two segments of the same proto-group could have moved in opposite directions: one toward Lake Maracaibo, and the other toward the eastern coast. The time of these movements remains to be determined. María E. Villalón (1987) proposed a ternary classification of Cariban languages and further suggested a division, in spatial terms, between nuclear and peripheral languages. This classification yields Eastern, Western and Northern units of the Cariban linguistic stock (Villalón 1987, p. 42, Fig. 11). While her proposal does not provide evidence for distinguishing the three areas, it does serve to illustrate a model of purported expansion by Cariban-speakers. A decade later, Gildea (1998) pointed out a series of possible shared innovations in phonology and inflectional morphology that would link the geographically neighbouring Cariban languages into a Venezuelan Branch. This branch would comprise a core group of languages including †*Tamanaku*, *Panare*, and the *Pemóng* Group, as well as several outliers, namely *De’kwana*, *Mapoyo*, *Yabarana*, *Chaima*, and †*Cumanagoto* (Gildea 1998). More recently, Gildea (2012) organized the ‘the modern Carib Family by degree of evidence for higher-level grouping’, subdividing the Venezuelan Branch in two macro-groups *Pemóng*-*Panare* and *Mapoyo*-*Tamanaku*. The former includes the *Pemóng* Group and the *Panare*, whilst the latter comprises the extinct †*Kumaná* (†*Chaima* and †*Cumanagoto*), *Mapoyo*/*Yawarana*, and †*Tamanaku*.

The archaeological perspective on the correlation between material culture and Cariban languages may well be buttressed by an example from northwestern Venezuela. In the foothills of the Sierra de Perijá, Arvelo (1987, 1991) noted the presence of pottery in the El Diluvio and El Zancudo styles (i.e., the Berlin tradition) dated to between 1350 BP (AD 600) and the Contact Period. The latter style features some characteristics of Valencioid pottery and was dated to between 1137 ± 62 BP and 1064 ± 60 BP (AD 813–886) (Sanoja 1969, pp. 90, 102). Considering the Cariban-speaking *Yukpa* oral tradition, Arvelo (1987, 1991) suggested that the penetration of these Amerindian groups into the Sierra de Perijá might have occurred, following long struggles, in the period 1150–950 BP (AD 800–1000) (Ruddle and Wilbert 1983). The *Yukpa* Group (*Yukpa* and *Yapréria*) was included in a so-called statistical ‘residue’ category of groups and languages for which the linguists are still in search of branches (Gildea 2012, Fig. 2). Nevertheless, the above-mentioned period coincides with the dispersion of the Berlin tradition groups (which encompassed the El Zancudo style), and in principle also coincides with the current distribution of the *Barí* people of Chibchan linguistic affiliation. As a result, the question arises whether the Berlin tradition is related to the Chibchan or Cariban groups or both. We would argue that the association between the El Zancudo ceramic tradition and Cariban-speaking groups seems more consistent, considering: (1) the oral history of the *Yukpa* (as noted by Arvelo 1987); (2) the proposed dates for the Berlin tradition expansion plus its possible Cariban relationship with the Valencioid westernmost expansion, as well as a possible association with Orinoquian groups; and (3) that in principle, the distributional coincidence between El Zancudo-style pottery and the contemporary *Barí* group (of Chibchan descent) merits further consideration. The exact distribution of the *Barí* at the time of European contact is unknown (see Gordones-Rojas 2007). Nevertheless, as interpreted by early colonial sources (Beckerman 1978), these people were apparently being dislodged from the La Grita area (Castillo-Lara 1998; Urbani and Viloria 2002) during the colonial period (Arcila-Farías 1967). This would prevent us making a connection between this Chibchan group and the El Zancudo-style pottery deposited in this area several centuries earlier. Therefore, Cariban-speakers who migrated into the Sierra de Perijá by 1150–1050 BP (AD 800–900) might have been typical Western Cariban-speakers as proposed by Villalón (1987; see also Tarble 1985), and at the same time makers of the El Zancudo pottery. Following Villalón’s contention, we argue that the above-mentioned evidence suggests the following: the same social and ecological pressures which might have prompted the migration of Cariban-speakers west by 1150–1050 BP (AD 800–900) might also have propelled other Cariban-speakers north from the Middle Orinoco at the same time.

## Conclusions

In this paper, we have employed the most recent data provided by archaeology and related disciplines conjoined with data collected decades ago in order to construct normative culture history. We note that the notion of ‘culture area’ still haunts our reappraisal of the ‘mobility’ of Cariban-speakers. Cultural homogeneity of archaeological traits, as discussed in this paper, has often been associated with territorial dominance by the bearers of a ceramic style or complex, or tradition. Linguistic dominance in the same area is often also assumed. In these conceptualizations, intergroup interactions between indigenous peoples have usually been conceived in the essentialized form of power relations, that is, dominance and subordination, despite the insufficiency, inadequacy, or simple unavailability of archaeological and linguistic data to support this conclusion. These interactions have not been considered to occupy a spectrum between war and peace; instead they have been shunted to the extremes. This is well illustrated by the case of the purported subjugation of the coastal villages of the egalitarian Ocumaroid by the inland Valencioid chiefdom (Sanoja and Vargas-Arenas 1974; Vargas-Arenas 1990). Simply put, archaeological data to support such a scenario is absent (Antczak 1999). A wide array of interactions, often falling between clear-cut war and peace, both among various groups of indigenous peoples and between these and Europeans after 1492, might well account for the archaeological data currently available. Such interactions may further be approached through the theoretical perspectives of, among others, acculturation, transculturation, ethnogenesis or hybridity (Ortiz 1995; Deagan 1998; Tarble de Scaramelli and Scaramelli 2011; Hu 2013; Liebmann 2013). Language undoubtedly played a variety of roles in intergroup mobility and exchange, as well as in collective remembering and social representations of history. It was but one of the social tools with which specific indigenous groups reconstructed their past and transmitted this knowledge to succeeding generations (cf. Liu 2012, p. 7). However, the nature and dynamics of these social phenomena largely remain to be disclosed through interdisciplinary investigations. Another pressing concern in future research is the systematic interdisciplinary assessment of the purported correlation between the archaeological radiocarbon dates from Orinoquia and the mega-Niño climatic events dated to c. 1500, 1000, and 700 BP (AD 450, 950 and 1250). Meggers (2001, p. 314, 1996) suggested that dramatic discontinuities observed in the regional cultural sequences (Saladoid, Barrancoid and posterior) may be due to ‘severe drought [that] would have depleted local subsistence resources and forced the members of the communities to disperse until conditions returned to normal.’ Some of these events may be causal to the mobility and restructuring of social and political organization of indigenous societies addressed in this paper.

Thus, in the preceding sections we have opened Pandora’s box. Multiple and diverse threads of potential interrelations among the purported Cariban-speaking bearers of the Arauquinoid and Valencioid cultures have emerged from it. As has been suggested by Hornborg (2005), some of the data reviewed in this paper can be construed to argue for the expansion of the Arawakan-speakers. Or it can be seen, perhaps, to signal linguistic and social exchange as well as adoption of new lifestyles by the people who already inhabited north-central Venezuela—rather than reflecting northward migration by Orinocans. The evaluation of such a possibility is pressing, especially while a dense net of intersocietal relations is emerging from the archaeological record of the late Ceramic Age in the study regions. In order to test the migration hypothesis thoroughly, interdisciplinary cooperation among the fields of archaeology, linguistics, history, ethnography, bioanthropology, ecology, and other life sciences is essential (Koch et al. 2014). A synergetic enterprise of this kind should be carried out under strict chronological control of an adequate number of representative samples. Although such a task is likely to be colossal, below we discuss some specific avenues of research that can be undertaken.

We explored and rethought the evidence for validating the traditionally favoured riverine route supposedly followed by migrants from the Middle Orinoco region to north-central Venezuela across the central *llanos.* We did this by suggesting the use of the Guárico, San José/Tacatinemo, and Manapire Rivers. Once in the north, the migrants could, alternatively, have used a terrestrial route, following the La Puerta-Villa del Cura-Cagua corridor. Indeed, this same terrestrial route could have been used by previous populations, but the archaeology is grudging on this matter. Although the initial step in accessing this route implies crossing the Cordillera del Interior mountain range, we have argued that this could well have been a worthwhile effort. Following this route, the migrants would have been enabled to avoid not only areas inhabited by potentially hostile Arawakan-speaking groups to the west, but also strenuous navigation upstream on small highland rivers. A systematic survey of the archaeologically unrevealed Cordillera del Interior may rule out or, conversely, support the above hypothesis. In the same vein, new radiocarbon dating may put the proposed date of 1150 BP (AD 800) as the beginning of the migratory movement to the test.

Provenance studies of statistically representative samples of both Valencioid and Arauquinoid wares are necessary to test their common origin. Provenance studies of Los Roques Archipelago and north-central Venezuelan mainland Valencioid and Ocumaroid pottery, especially the figurines, have already been performed (Kasztovszky et al. 2004; Sajó-Bohus et al. 2005, 2006). However, the ‘early’ traits of Valencioid pottery should be understood and fixed since currently only a distinctively ‘mature’ Valencia style is known from the Lake Valencia Basin. The isolation of early Valencioid/Barrancoid hybrid pottery resulting from the processes of transculturation would also be expected. Petrographic and ceramological analyses would strengthen the conclusions drawn from chemical and mineral compositional analyses. Imagery and body adornments may not only be formally compared, but also used to ontologically explore commonalities in both cultures as regards interrelations with other-than-human beings (Antczak and Antczak 2017a).

The potential for comparative bioanthropological analyses seems to be enormous. Nevertheless, the available samples decidedly favour the Valencioid side: the museums in Valencia and Maracay are repositories of the most numerous Amerindian skeletal remains in Venezuela. Bioanthropological research may permit the identification and examination of the health status of phenotypes, including analysis of the intentional skull modification amply reported in Valencioid populations (Jahn 1932; Requena 1947; de Arechabaleta 1979; Fossi et al. 1995; Bonilla and Morales 2001). Perceived changes in diet between the Orinocan and northern populations may successfully be addressed by isotopic analyses. At least after 1050 BP (AD 900), Valencioid peoples became increasingly exposed to a diet rich in proteins of marine origin. From 750 BP (AD 1200) onwards they became receptors and consumers of a great volume of queen conch meat (Antczak and Antczak 2006, 2008). Archaeogenetic analyses may be crucial in tracking down affinities between Cariban-speaking Orinocans, their Valencioid relatives, and north Venezuelan Arawakan-speakers. The abundance and diversity of burials, especially in the Lake Valencia Basin, coupled with good preservation and rich burial offerings, present a unique opportunity to study mortuary practices, paleodemography and status related to sex and age. Burials in urns, an outstanding form of internment in the Valencia region (Requena 1932; Kidder 1944; Peñalver 1965, 1967, 1971), have also been associated with Arauquinoid expansion (Zucchi 1976; Gassón 2002).

Another venue for future research is related to rock art which is prominent in both study areas (Scaramelli 1992; Rivas 1993; Delgado de Smith et al. 1999; Antczak and Antczak 2007; Páez 2007, 2011). The evaluation of possible connections between the northward spread of the rock art traditions and the Arauquinoid expansion seems especially promising (Greer 1995, p. 303, Fig. 36). Comparative analyses may also address the broader context of the creation and maintenance of socio-cosmological landscapes in both regions (Scaramelli and Tarble 1996; Tarble and Scaramelli 1999; Vidal 2000; Zucchi et al. 2001). Here, the applicability of Ernst Halbmayer’s broader conception of the embedding of time and space in Amerindian cosmologies and social organization may prove useful for future research. This is especially interesting because it has already been well-exemplified in the case of the socio-cosmological landscapes of the Cariban-speaking Yukpa from Venezuela and Colombia (Halbmayer 2004, 2012). Visual differences between late pre-Hispanic Amerindian imagery in north-central Venezuela and the neighbouring macro-regions have been pointed out (Delgado 1989; Díaz Peña 2006; Caputo Jaffé 2013). However, the possible interrelation between these varying phenomena and large migratory movements, as well as the active role of this imagery in forging the sociomaterial worlds of indigenous peoples, have not yet been researched by archaeologists (but see Antczak and Antczak 2006, 2016).

Landscape engineering, especially mound building, as well as the signatures left in the landscape by purportedly intensive horticulturalist practices, are ripe for geomorphological, geochemical, and archaeobotanical research. In addition, a large-scale archaeological survey is still needed in the central *llanos*. At the moment, the research agenda for archaeology in the Venezuelan *llanos* is concentrated both in the western part of this macro-region (e.g. Gassón and Rey 2006; Gassón 2007b; Rey-González 2007; Spencer et al. 2014), and in the eastern *llanos* (e.g. Rodríguez and Navarrete 1995; Navarrete 2000; Rodríguez-Yilo 2001).

The Arauquinoid entered the resource-rich Lake Valencia Basin through the migration path discussed in this paper, even though such movement could have been met with resistance from local populations and would initially have decreased Arauquinoid population growth. Although future research may inform us of more nuanced mechanisms for these early contacts, judging from the archaeological record, the Arauquinoid migrants became highly successful in settling the north-central Venezuelan highlands. While taking possession of the Lake Valencia Basin by 1150 BP (AD 800), these Cariban-speaking groups seemingly pushed the local Arawakan-speaking residents to the Caribbean coast. After the apparent initial seclusion of the migrants in the confines of the Lake Valencia Basin, by the end of the first millennium AD they were already ‘released from the proximity’ (see Knappett 2014, p. 4705) and had increasingly been forged by the influx of diverse new materials, technologies, and information from distant places (Antczak and Antczak 2015a). After 950 BP (AD 1000) they had thrust themselves towards the Caribbean coast as well and developed large-scale queen conch (*Lobatus gigas*) exploitation on the off-shore oceanic islands after 750 BP (AD 1200). Concomitantly, they created the Valencioid Sphere of Interaction that assured the long-lasting success of this conspicuous island enterprise (750–450 BP; AD 1200–1500). Valencioid quotidian practices and identities were constantly re-constructed, amended, and transformed by their interactions with ‘things’ around them, including other human groups, other-than-human beings, the surrounding environments, and the interface of these dynamics with their individual and social memory. It is challenging to elucidate not only how the sphere was born and maintained on a regional scale, but also how it animated the inter-ethnic, inter-cultural and inter-lingual reciprocal actions entwined with far-reaching interregional interdependence systems at meeting points in the Orinocan south, Andean west and Caribbean north (Vargas-Arenas et al. 1984; Molina 1985; Biord 1985, 2005; Antczak and Antczak 1999, 2006; Boomert 2000; Heinen and García-Castro 2000; Sýkora 2006; Rodríguez-Ramos and Pagán-Jiménez 2006; Biord and Arvelo 2007; Hofman et al. 2007, 2008, 2014; Curet and Hauser 2011). We consider that the modelling of the processes anticipated in this paper using agent-based models and network theory may prove successful in disentangling that which is still tangled and hidden in the archaeological data. However, if future research proves able to validate the Orinocan origin and Cariban linguistic affiliation of the bearers of the Valencioid culture, that culture will be considered one of the most dynamic sociopolitical Cariban-speaking entities in northeastern South America and the insular Caribbean on the eve of European Conquest.
